# Honeycombs – their variety, topology and symmetry

**DOI:** 10.1107/S2053273325000889

**Published:** 2025-02-14

**Authors:** Zbigniew Dauter, Mariusz Jaskolski

**Affiliations:** a Center for Structural Biology National Cancer Institute Frederick MD USA; b Department of Crystallography, Faculty of Chemistry Adam Mickiewicz University Poznań Poland; c Institute of Bioorganic Chemistry Polish Academy of Sciences Poznań Poland; Universidad del País Vasco, Spain

**Keywords:** honeycombs, wax economy, layer symmetry, Dirichlet domains

## Abstract

The double-layer honeycomb with hexagonal cells, three rhombic faces between the two layers and *p*3*m*1 layer space-group symmetry, used universally by honeybees, is often considered to be the most efficient (from the point of view of wax economy) and the only honeycomb manufactured by bees. However, another variant of a symmetric and periodic double-layer hexagonal honeycomb with two hexagons and two rhombi between the two layers and slightly better wax economy was discovered theoretically in 1964 by Fejes Tóth and found in nature some years later. The present work shows that there is yet another possibility, with the interface formed by one hexagon and two quadrangles, in addition to the trivial case with flat hexagonal cell bottoms and very poor wax economy. Moreover, we demonstrate that the geometry of the Fejes Tóth honeycomb can be optimized for even better wax economy. All the theoretical honeycomb types are derived using the principle of Dirichlet-domain construction and shown to have more and less symmetric variants. Wax economy is calculated for each case, confirming that indeed the modified Fejes Tóth honeycomb is the most efficient, while the trivial flat-bottom case is the least.

## Abstract 2

Several types of possible hexagonal double-layer honeycombs are described and analyzed from the point of view of their topology, symmetry and wax economy, including the newly discovered honeycomb with minimal use of wax.

## 1. Introduction

The elegant symmetry and construction principle of the double-layer honeycomb has fascinated people for centuries. In his famous book *De Nive Sexangula* (*The Six-Cornered Snowflake*), Johannes Kepler (1611 ▸, 1966 ▸) dissected the trigonal interface between the two layers and associated it with the closest packing of identical spheres (Dauter & Jaskolski, 2014 ▸). Our own interest in the structure and symmetry of honeycombs is also a spinoff of our work on sphere packing (Jaskolski *et al.*, 2025 ▸).

**Figure 1:**
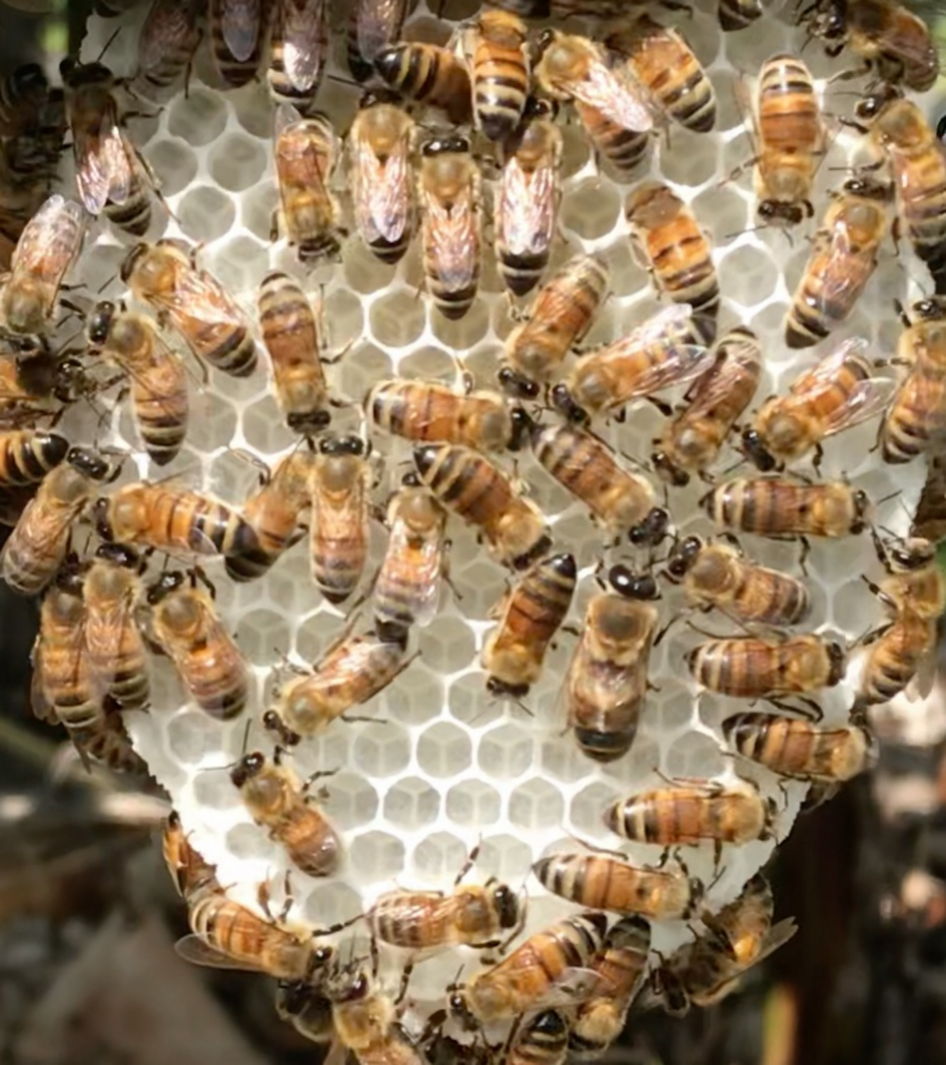
Honeybees building a honeycomb. Courtesy of Laryssa Kwoczak from https://www.beekeepingmadesimple.com/.

The classic honeycomb, constructed with natural beeswax and adopted almost universally by most honeybees, consists of two layers of hexagonal cells, which are used by the queen to lay eggs, and in which the hatched larvae and then pupae are appropriately fed (with the purpose of nurturing workers, drones or a new queen) by the worker bees (Fig. 1 ▸). (In fact, drones are raised in larger and less regular cells, while the queen cell is separate and very large.) After full metamorphosis, the empty cells are used for storing honey and pollen, *i.e.* they function as a food depot for the bee colony. Opinions are still divided as to how the bees actually manufacture their combs, but the prevailing notion is that they start from some less regular patterns, such as a bubbly foam-like structure (Weaire & Phelan, 1994 ▸) or a hexagonal lattice of cylindrical cells (Nazzi, 2016 ▸), which are gradually converted into a regular lattice of hexagons due to the physical properties of beeswax.

**Figure 2:**
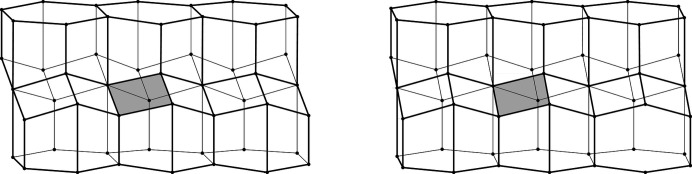
Stereoview of a fragment of the classic two-layer hexagonal honeycomb with 444 topology of cell bottoms. There are two layers of identical cells, here shown as an upper and lower layer (in reality they are left and right layers of a vertical honeycomb). The designation 444 denotes three quadrangles (here rhombi) forming a cell floor (or ‘ceiling’ in the lower layer). One of the rhomboidal faces of the central lower cell is shaded gray. The bottoms of the cells from the two layers fit perfectly together, forming a corrugated interface.

The two identical layers of a honeycomb are packed back-to-back with their bottoms at the interface. The bottom of each cell is not flat but is constructed as a convex pyramidal dome from three identical rhombic faces, arranged according to 3*m* symmetry. The topology of the trigonal bottom is 444, meaning that it is formed from three quadrangles. The trigonal bottoms of the two layers nest tightly together, forming a corrugated sheet of rhombic faces, which is the ideal, single-walled interface of the two-layer structure (Fig. 2 ▸). In other words, each rhombic face of the trigonal bottom is shared by two cells, one from each layer, and the three rhombic faces of a cell from one layer are shared by three adjacent but different cells from the opposite layer.

Since the production of wax is metabolically costly for the worker bees, the principle of honeycomb construction may be to cover a given volume with the minimal amount of wax. It has been intuitively felt that such a construction should correspond to the most efficient use of space and to the most economical (*i.e.* minimal) use of wax. However, in 1964 the Hungarian mathematician László Fejes Tóth (FT hereafter) realized that a similarly compact interface may be constructed with different cell bottoms, having 6464 topology, *i.e.* formed from two hexagons and two quadrangles. Even more importantly, FT calculated that the wax economy would be ∼0.35% better than in the classic 444 honeycomb. FT’s discovery turned out to be more than a mere mathematical curiosity when beekeepers in China found a strain of bees that actually use the 6464 principle for the construction of their honeycombs (Yang *et al.*, 2022 ▸).

In the present paper, we have revisited the honeycomb, asking if the two known honeycomb principles (444 and 6464) are all the possibilities of a consistent, symmetrical and periodic two-layer interface of hexagonal cells. We found that there is indeed yet another topology possible, denoted 644, in addition to the trivial case 6 of flat (hexagonal) bottoms, and that in each of the four topologies there are more and less symmetric variants, in analogy to the special and general positions of crystallographic space groups.

In addition, our analysis shows that the FT 6464 honeycomb can be geometrically modified to obtain a honeycomb with even better wax efficiency, 0.15% more economical than the FT variant and 0.50% better than the tri-rhombic 444 variant.

We derived each of the possible honeycomb topologies from the packing principle of Dirichlet domains and calculated the wax economy in each case. Moreover, we analyze the symmetry of each variant and describe it in terms of the layer space groups known in crystallography (Kopský & Litvin, 2006 ▸).

## 2. Geometric construction of the honeycombs

**Figure 3:**
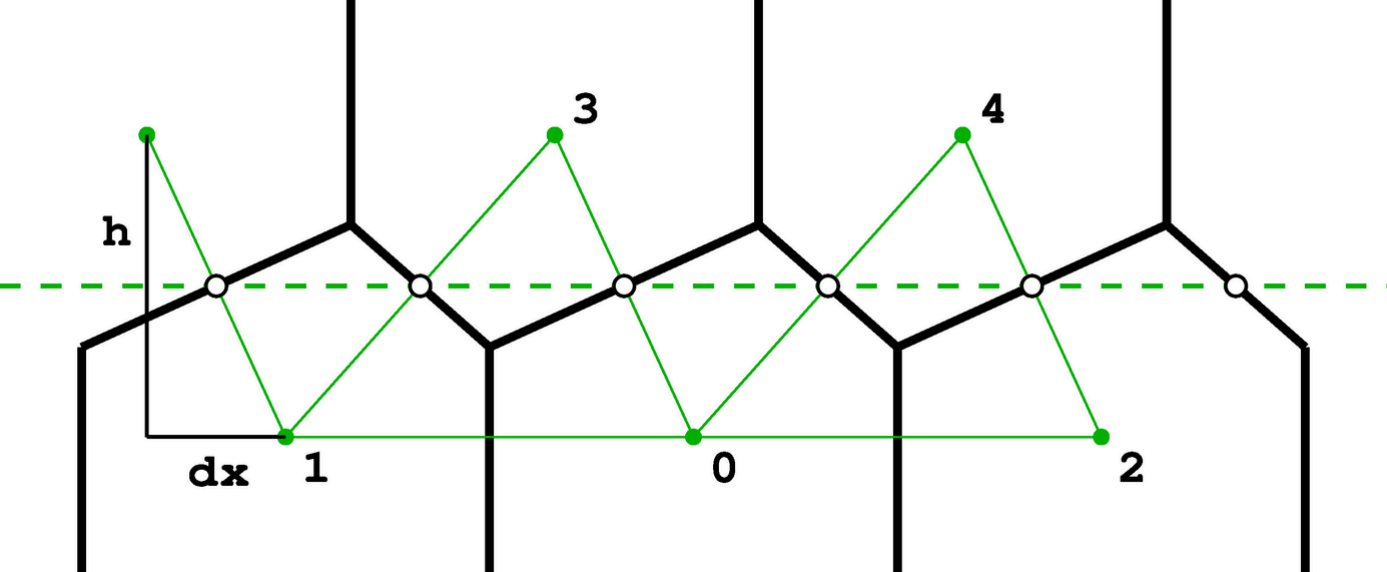
Construction of a 2D honeycomb. Each honeycomb edge (black) is perpendicular to a line (thin green) joining two neighboring generating points (green dots), equally spaced in two parallel rows on both sides of the mean honeycomb plane (dashed green line) passing through the centers of symmetry at mid-points of the bottom edges of each cell. The vertical edges are of arbitrary height, but they all reach the same level at the honeycomb outer base (where the openings of the cells are).

As mathematical models, honeycombs can be built using a procedure analogous to the construction of Dirichlet domains, *aka* Voronoi polyhedra (Nowacki, 1935 ▸, 1976 ▸). Fig. 3 ▸ presents a 2D version of this procedure. It starts with two parallel rows of equally spaced points called generators, marked green and numbered 0–4 in Fig. 3 ▸. The rows are at a distance *h* and have a relative shift of d*x*. Each of the generator points is surrounded by (black) line segments that are perpendicular at half-length to the vectors joining these points with their neighbors (thin green lines). The black lines (planes in 3D) would mutually intersect forming a well defined convex polygon (polyhedron in 3D). In the construction of true Dirichlet domains (*i.e.* for an infinite array, or lattice, of generators) such lines (planes) would enclose a complete and identical polygon (polyhedron) around each generating point. Since in the present construction a neighboring row of points is only on one side, the resulting cells are open at the opposite side. Each thus-obtained cell has two parallel, vertical edges resulting from inter-generator contacts within the same row, as well as some other edges at the bottom, corresponding to neighbors from the opposite row. [The length of the vertical segments (or cell walls) is arbitrary but they all have to reach to the same hexagonal base plane at the cell openings. In nature, cell depth (or cell wall height) may vary with bee species.] The arrangement (shape) of these bottom edges depends on the particular values of the *h* and d*x* parameters. Such a 2D honeycomb has centers of symmetry at mid-points of the line segments at the cell bottoms, located at their intersections with the mean plane (dashed green line) passing through the honeycomb.

In 3D honeycombs, instead of two rows of generating points, there are two parallel layers of points arranged on a pattern of symmetric hexagons. Again, vectors between these points and their neighbors define intersecting planes, which eventually form the ‘half-domains’ (in Dirichlet sense), open at one side. The resulting cells will always have six vertical faces arising from contacts with points within the same layer, forming a hexagonal prism, as well as some faces at the bottom, between points from the two opposite layers. Of course, since all the generating points are symmetrically equivalent, all cells must be identical and the whole honeycomb arrangement has well defined symmetry, corresponding to one of the layer space groups (Kopský & Litvin, 2006 ▸). The exact shape of the cell in 3D depends on the distance *h* between the two layers of generating points and on their relative parallel shifts d*x* and d*y*. There is always a center of symmetry at the center of each bottom face and located at the mean honeycomb plane. The honeycomb, therefore, always has at least the layer space group *p*1. For some particular d*x* and d*y* values, however, the symmetry may be higher (see Section 4).

Table 1 ▸ illustrates certain aspects of honeycombs with various d*x*, d*y* layer shifts and, therefore, with various topology and symmetry. All the diagrams in Table 1 ▸ assume that the parameter *h* is selected to minimize the total area of all honeycomb cell faces for given values of d*x* and d*y*. The blue outline in column (1) of the d*x* = 0, d*y* = √3/2 honeycomb (second row) corresponds to the newly discovered (see Section 5) most economical honeycomb, while the black color corresponds to the FT honeycomb that is not optimal in this respect. Geometrically, these two honeycombs differ by a 2.8° rotation of the hexagonal bottom faces around their horizontal (long) diagonals and a concomitant rotation of the rhombic faces.

## 3. The topology of possible honeycombs

**Figure 4:**
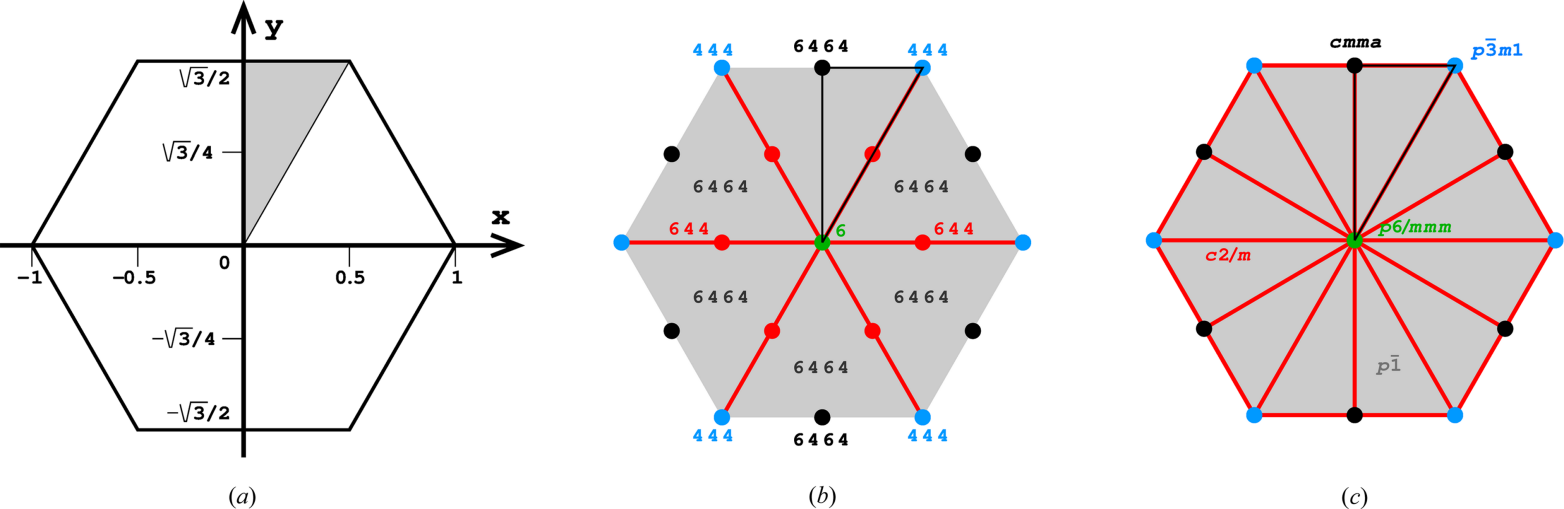
Diagrams illustrating the coordinate system with the asymmetric unit in gray (*a*), as well as the topology (*b*) and layer space-group symmetry (*c*) of the honeycombs in relation to the location of the site where the center of an upper-layer cell projects onto the mean plane of the lower layer. See text for explanation.

Fig. 4 ▸(*a*) shows the coordinate system used for the description of the honeycomb geometry. It is assumed that the edge of the hexagonal honeycomb cell cross section has unit length. Panels (*b*) and (*c*) show a projection of the lower honeycomb cell on the honeycomb mean plane. The points and lines, shown in different sets of color in these panels, mark the sites where the center of the upper cell will project onto this plane according to different d*x* and d*y* shifts and different topologies.

There are four distinct honeycomb topologies, designated using the ‘vertex count’ notation, as shown in Fig. 4 ▸(*b*). ‘6464’ means that each cell has four faces at the bottom in the circular order of a hexagon, tetragon, hexagon and tetragon. ‘444’ describes the bottom faces of three tetragons *etc*. Within the regions marked by different colors, the topologies are the same but, of course, the individual shapes of the honeycomb faces may be different. These shapes will also depend on the parameter *h*, which defines the distance between the planes of the Dirichlet centers (generators) of cells in the two honeycomb layers. The topology, however, does not depend on *h*.

In general, the whole hexagonal region, shown in gray in Figs. 4 ▸(*b*), 4 ▸(*c*), has the 6464 topology and *p*1 layer symmetry for the majority of honeycomb arrangements, except certain specific lines and points marked in different colors. In Fig. 4 ▸ and in all subsequent figures, as well as in the trigonometric calculations in the supporting information, it is assumed that the horizontal direction is along *x* and the vertical direction is along *y*, as marked in Fig. 4 ▸(*a*). The asymmetric unit (ASU) of the hexagons in Fig. 4 ▸ is the triangle delimited by the thin black lines, covering a region with the coordinates of 0 ≤ *x* ≤ 1/2 and 0 ≤ *y* ≤ *x*√3.

For the calculations and all figures, it is assumed that the relative shift of d*x* = d*y* = 0 of the two honeycomb layers corresponds to the trivial situation where the center (*i.e.* generating point) of one layer is exactly above the center in the other layer at a distance *h*. Such a honeycomb, with a flat hexagonal bottom face and 6 topology, corresponds to the green vertex of the ASU triangle.

The blue dot at d*x* = 1/2, d*y* = √3/2 corresponds to the classic tri-rhombic honeycomb with 444 topology. The black dots at mid-points of the hexagon edges at d*x* = 0, d*y* = √3/2 mark the 6464 topology with the orthorhombic *cmma* symmetry. They correspond to both, the FT and the ‘best’ honeycombs.

The long red diagonal of the hexagon in Fig. 4 ▸(*b*) with the equation d*x* − d*y*/√3 = 0, including the red point at its center, represents the 644 topology. The remaining, gray region of Figs. 4 ▸(*b*), 4 ▸(*c*) corresponds to the 6464 topology.

## 4. Honeycomb symmetry

The different colors in Fig. 4 ▸(*c*) correspond to different symmetries of the honeycombs, obtained when cells of one layer, shown in projection on the mean honeycomb plane, are translated relative to cells in the complementary layer. Again, the gray color corresponds to mapping of honeycombs with *p*1 symmetry (and 6464 topology). The honeycomb corresponding to the black point at the center of the outer hexagon edge also has this topology, but represents the FT and the ‘best’ cells with the layer space group *cmma*. All three red lines, marked in Fig. 4 ▸(*c*) as borders of the ASU triangle, correspond to honeycombs with the *c*2/*m* layer space group. On each line, however, the monoclinic twofold axis has different orientation, perpendicular to each of the ASU edges. These lines also represent two different topologies, 644 and 6464.

The blue points at d*x* = 1/2, d*y* = √3/2 correspond to the classic tri-rhombic honeycomb with *p*3*m*1 layer symmetry, while the green point at the hexagon center, *i.e.* with perfect overlap of flat-bottom cells from the two layers, corresponds to the *p*6/*mmm* layer space group.

Symmetry of the honeycomb types also dictates the number of ways in which the two layers can be matched. For example, with the 3*m* point symmetry of the classic 444 honeycomb cell, one of the layers will fit the other in three orientations, differing by 120° rotations as well as after three mirror reflections of this point group. The trivial honeycomb 6 with 6/*mmm* point symmetry allows six matching orientations, differing by 60° rotations and 6+1 mirror reflections. For the *mm*2 and 2/*m* point symmetries the number of mutual orientations of the two honeycomb layers also reflects all the symmetry operations of these layer space groups.

## 5. Wax economy of the honeycomb variants

The amount of wax needed to build a honeycomb is proportional to the total area of all faces of the honeycomb cell. As mentioned in the *Introduction*, it had been thought for a long time (and often still is) that the classic tri-rhombic 444 honeycomb represented the most economical (*i.e.* requiring the least amount of wax) honeycomb architecture. It was, therefore, a big revelation, when Fejes Tóth (1964 ▸) demonstrated mathematically that a more economical honeycomb (the symmetric 6464 variant) is possible. A strong real-life confirmation for his findings was provided by Yang *et al.* (2022 ▸), who reported that such natural honeycombs are indeed built by some bees in China. The relative gain of the FT honeycomb over the classic 444 version was calculated as less than 0.35% of the reference area of the hexagonal cell cross section (Fejes Tóth, 1964 ▸).

**Figure 5:**
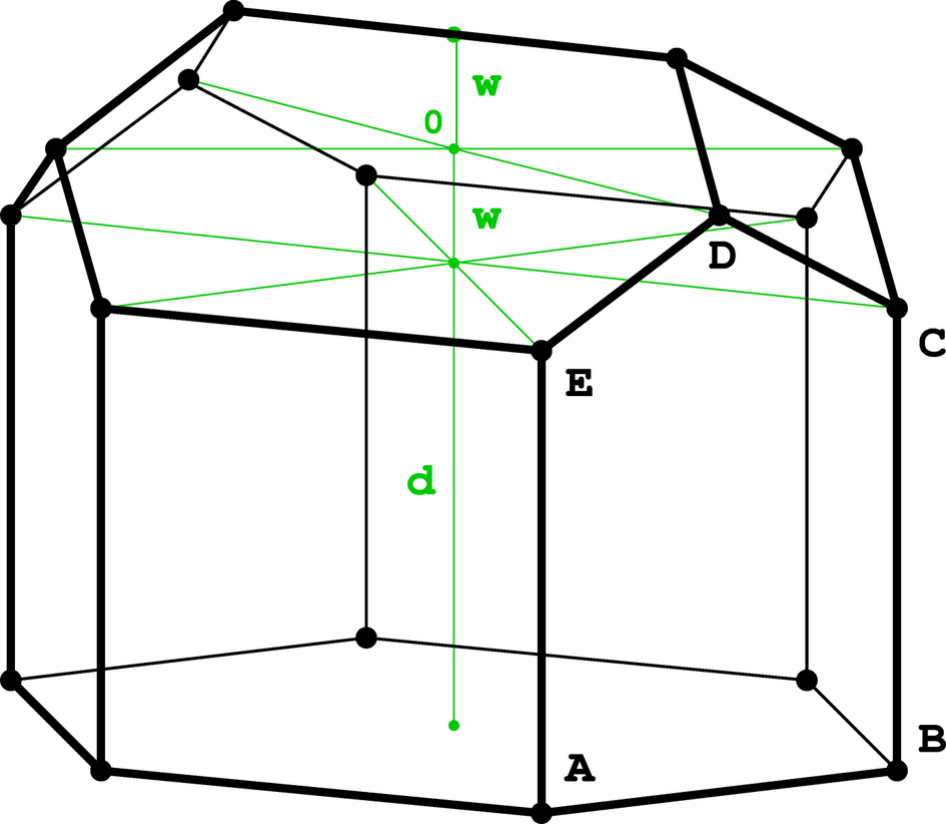
Illustration of a honeycomb cell with d*x* = 0, d*y* = √3/2, showing how the positions of the cell-bottom vertices (here shown at the top) are related to the parameter *w*.

**Figure 6:**
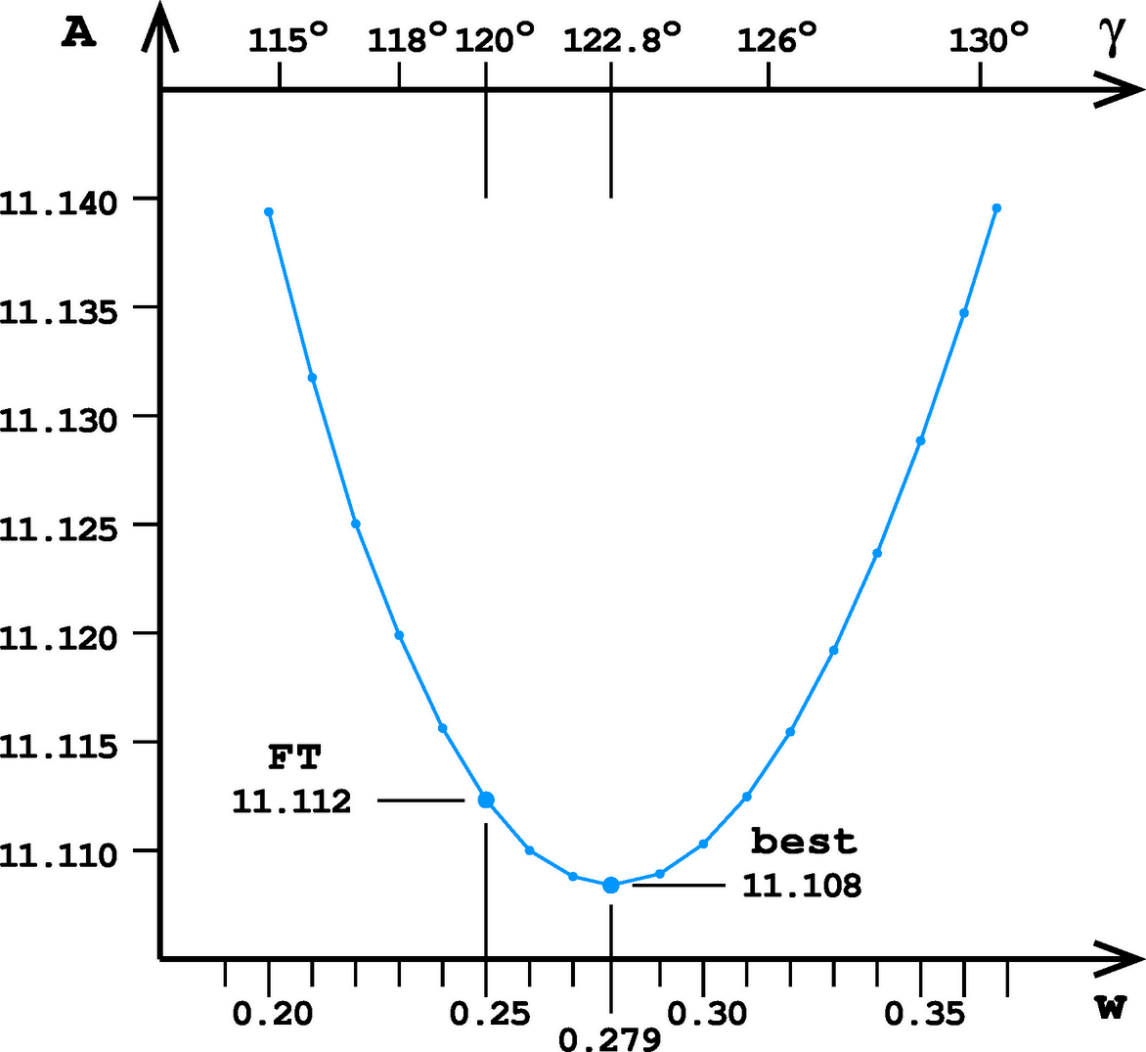
Dependence of the total cell face area *A* of the honeycomb on the distance *w* from the cell-bottom middle vertices to the honeycomb mean plane (Fig. 5 ▸). This graph is relevant for honeycombs with d*x* = 0, d*y* = √3/2, including the ‘best’ and FT versions. The angle γ on the alternative abscissa shows the corresponding dihedral angle between the cell floor hexagon and its adjacent prismatic face of the 6464 cell. The two larger points marked on the plot correspond to the total cell face areas *A* of the FT and ‘best’ honeycombs.

Here we show that the cell surface area economy can be further improved, beyond the FT gain, to 0.50%, if the tilt of the hexagonal faces of the FT honeycomb is increased by 2.8° [Fig. 3 ▸(*a*)]. The optimal geometry can be derived by assuming the unit length of the hexagonal base edge and calculating the total area of all cell faces as
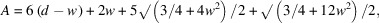
where *d* is the half-width of the double-layer honeycomb and *w* is the distance of the bottom vertices from the mean plane of the 6464 honeycomb, as shown in Fig. 5 ▸. The four components of the formula for *A* designate, respectively, (i) the six rectangular ABCE segments of the faces of the hexagonal prism, (ii) the four CDE triangles, (iii) the two cell bottom hexagons and (iv) the two cell bottom rhombi. The extremum of the *A*(*w*) function is found in the zero point of the first partial derivative ∂*A*/∂*w*, and is additionally confirmed as a minimum by plotting the *A*(*w*) function (Fig. 6 ▸). The minimal area occurs at *w* = 0.279214, which is different from the value of 0.25 assumed by FT.

The exact dimensions of the honeycomb cells vary among different bee species, but also between individual cells of the same honeycomb (*e.g.* Hailu & Biratu, 2016 ▸; http://www.dave-cushman.net/bee/cellsize.html; https://beeinformed.org/2017/11/03/comb-management-part2-comb-size/). The width of the worker bee honeycomb cells varies from 4.5 to 5.5 mm between different species and can also vary up to 10% within one honeycomb. The cell depth usually measures between 9 and 12 mm. Our interpretation of the honeycomb geometry is to some extent idealized by the assumption that all cells are identical and symmetrically arranged in two opposite hexagonal layers. In the numerical calculations presented in Table 2 ▸ and in the supporting information, it was somewhat arbitrarily assumed that the depth of the honeycomb cell, *i.e.* half-thickness of the whole two-layer honeycomb, is *d* = 1.5 times the length of the edge of the hexagonal cell base. (We note that the relative wax economy is independent of the arbitrary value of *d*. For real-life honeycomb cells one could assume *d* ≃ 3.5 times the length of the edge of the hexagonal cell base.) All values quoted in Table 2 ▸ and in the supporting information are obtained for the optimal values of the *h* parameter that minimize the total face area of the honeycomb cell preserving the same values of d*x* and d*y*, with the exception of the FT case, where the total area is not optimized with respect to *h*.

**Figure 7:**
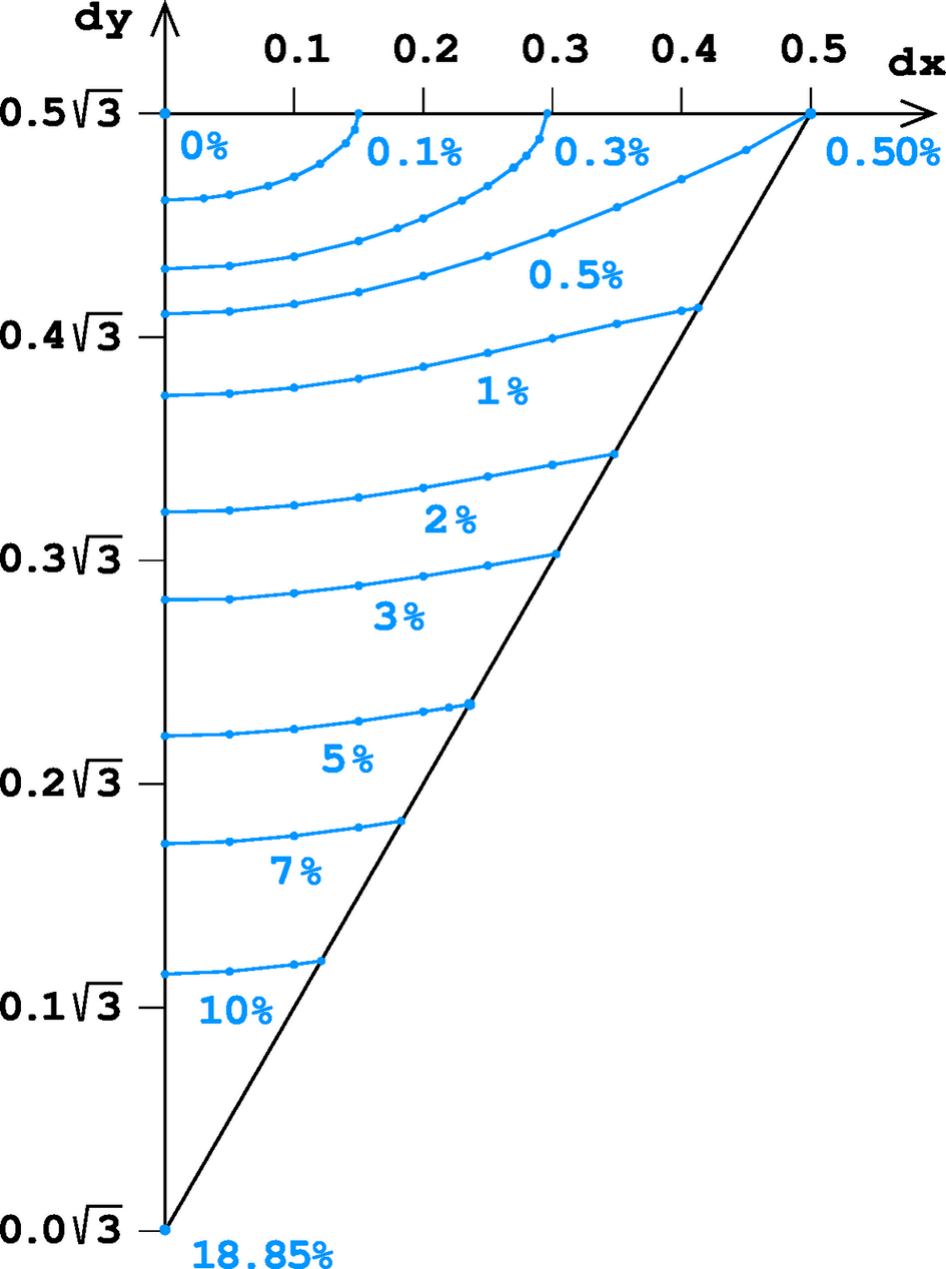
A diagram showing the difference between the total area of honeycomb faces (with optimized *h* values) and the area of the economically ‘best’ reference honeycomb, expressed in % of the hexagonal cell base area, for the ASU region marked in Fig. 4 ▸. The blue dots are for exactly calculated values and the lines show interpolated values.

Table 2 ▸ presents the area differences relative to the optimal value of the ‘best’ 6464 honeycomb, expressed in % of the hexagonal cell base area. These values are relative and do not depend on the arbitrary cell depth *d* parameter. These results are also presented in graphical form in Fig. 7 ▸.

It is evident from Fig. 7 ▸ that the honeycomb surface area is much more sensitive to the shift along *y* than along *x*. The difference between the classic and ‘best’ honeycombs is only 0.50%, and is achieved at d*x* = 1/2 (maximum possible) but at the same d*y* = √3/2 value. On the other hand, the difference is 18.85% for two honeycombs (‘best’ 6464 and trivial 6) with the same d*x* = 0 but shifted by √3/2 (maximum possible) along the *y* axis.

**Figure 8:**
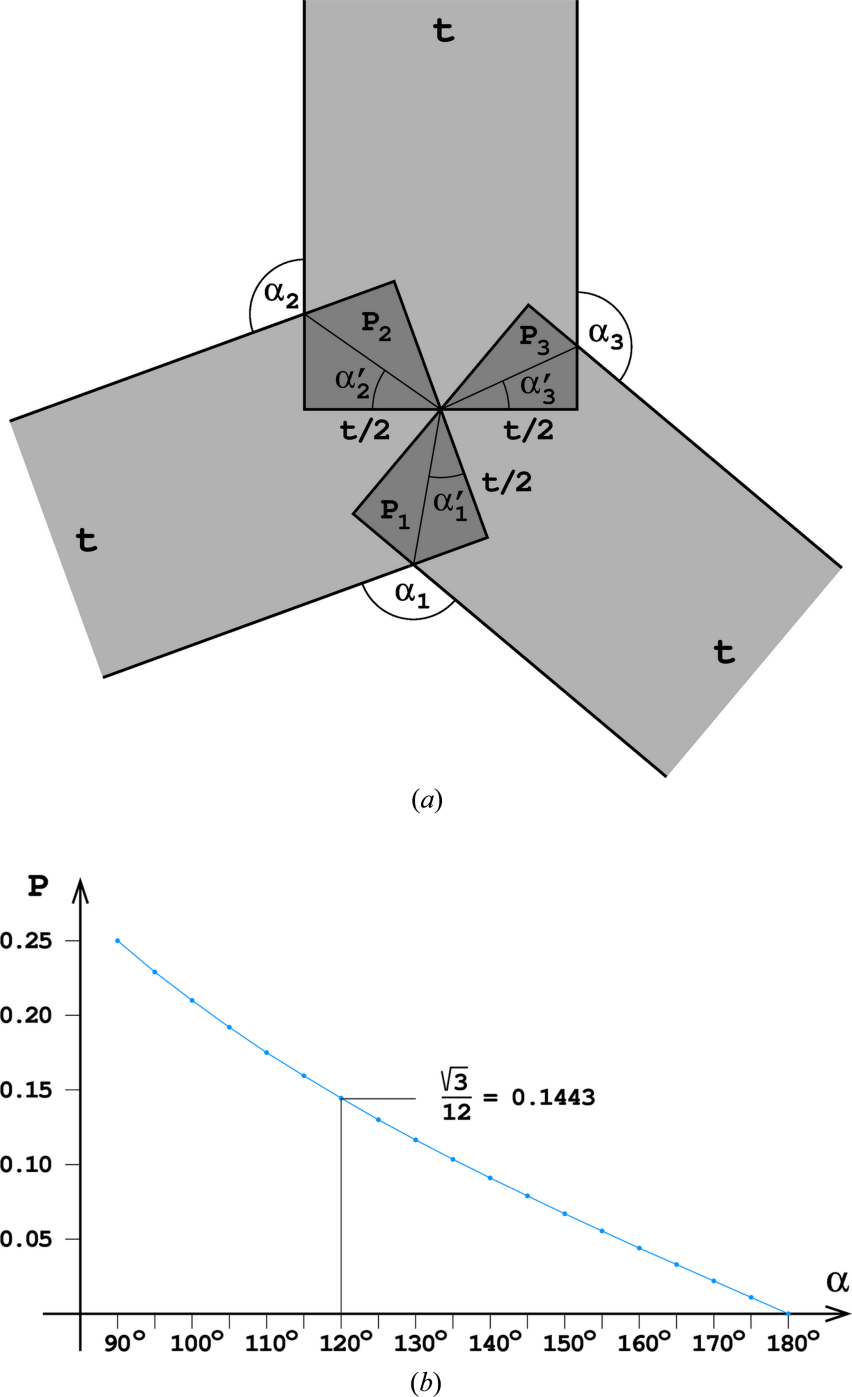
(*a*) Cross section of a junction between three honeycomb faces, each of thickness *t*, inclined at different angles to each other, with the darkened areas showing the surplus regions [*e.g. P*_1_ = *t*^2^/4 tan (90° − α_1_/2) *etc.*] where wax is saved. (*b*) Diagram showing the surplus area *P* at an edge formed by two honeycomb faces, in relation to the dihedral angle α between these faces. The values of *P* are plotted as fractions of *t*^2^.

However, consideration of honeycomb face areas without taking into account their thickness reflects an idealized case. In reality, the amount of wax also depends on the ways in which the faces join each other. Excepting the very border of the honeycomb, all edges formed by meeting faces are three-way junctions, as illustrated in Fig. 8 ▸(*a*). Likewise, all vertices are formed by at least six faces. The surplus (saving) area of wax at the edge formed by two faces of thickness *t* meeting at an angle α is *P* = *t*^2^/4 tan (90° − α/2). The total amount of wax is proportional to the area of all faces *A* times half the width of the cell face, *i.e.* ∼*A* × *t*/2. The surplus of wax at the junction of two faces is proportional to the edge length *l* times the square of the face half-width, *i.e.* ∼*l*(*t*/2)^2^. A similar effect is present at vertices, where six or more faces meet. However, here the surplus is proportional to the cube of the face half-width, *i.e* ∼(*t*/2)^3^. Assuming, as in the calculations of Table 2 ▸, that the thickness of the cell faces *t* is 10% of the cell base edge (*i.e. t* = 0.1), we get an estimate of vertex saving that is an order of magnitude smaller than the effect of the edges. We, therefore, neglect the influence of the vertices in the calculations presented in Table 2 ▸. The fraction of the surplus area relative to the *t*^2^ cross section at a cell edge formed by two faces joined at an angle α is presented in Fig. 8 ▸(*b*).

The amount of wax per one honeycomb cell is, therefore, calculated according to the following formula:
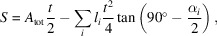
where the summation is over all cell edges of length *l*_*i*_ and internal angles α_*i*_, as listed in the supporting information for all the honeycombs under discussion. The Δ*S*/*S* column of Table 2 ▸ gives the relative saving of wax, shown as a fraction of a slab of wax (of amount *S*) of thickness *t*/2 and area equal to the cell base. It is a general conclusion that the more faces and edges there are at the cell bottom, the better the wax economy.

Superficially, it might appear that the honeycomb cell volume depends on its bottom topology. However, this is not the case. If we have a double-layer honeycomb of 2*d* thickness with hexagonal cells with base edge of unit length, then no matter how the interface between the identical layers is constructed, the cell volume will remain the same.

The supporting information presents auxiliary figures and detailed calculations of wax saving in different honeycomb configurations. It also recapitulates the FT-style derivation of the 6464 and 444 topologies.

## 6. Conclusions and outlook

The four topologies (444, 6464, 644, 6), and their less/more symmetric variants, of two-layered honeycombs are indeed all the possibilities of symmetric and periodic arrangements of regular hexagonal cells. However, this does not exhaust all the possibilities in general, because there might be, in analogy to periodic tiling of the 2D plane, cells shaped as triangles, general parallelograms, rectangles or squares. Such shapes were beyond the present analysis but might be the subject of a separate study. Moreover, one could also consider aperiodic tiling of the 2D plane with identical polygons, such as in the example of the einstein aperiodic monotile (Smith *et al.*, 2024 ▸), to which the third dimension would be added by (i) creating cells along the plane normal, (ii) ending them with an appropriate bottom (the same einstein monotile in the trivial case), and (iii) matching two such layers back-to-back across the common interface formed by the interweaving bottoms. Analysis of such an aperiodic honeycomb would be a tall order, however.

Finally, we note that beekeepers often prompt honeycomb making by inserting in the beehive frames artificially made ‘stump’ honeycombs, consisting essentially of the trigonal interlayer sheet with very short stumps of the prismatic cells, which serve as a template for worker bees. Such artificial honeycombs could be printed according to the optimal FT geometry, allowing the beekeeper (but not the bees!) to save a little bit on the wax material. It would be interesting to see how the bees would treat such modified honeycombs.

## Supplementary Material

Supporting information. DOI: 10.1107/S2053273325000889/uv5034sup1.pdf

## Figures and Tables

**Table 1:** The honeycomb variants discussed in this work, defined by the d*x*, d*y* shifts in column 0 The honeycomb features are presented in consecutive columns as follows: (1) a single honeycomb cell; (2) two pairs of cells from the two layers, showing how their bottoms fit together; (3) projection of one upper-layer cell (red) on several lower-layer cells (blue), with the Dirichlet-domain generating points in green (for the lower cells) or black (upper cell), and with auxiliary vectors between those points (green dashed lines) that generate the bottom faces of the red upper cell; the centers of symmetry are marked as small black circles and the black lines mark the reference frame for the coordinates of the vertices of the red cell (*x* axis horizontal, *y* vertical); the d*x*, d*y* shifts are marked explicitly in rows 3 and 7; (4) projection on the mean plane of a honeycomb fragment with several upper- and lower-layer cells, with all edges and vertices above the mean plane in red and all edges and vertices below that plane in blue; the vertices and edges lying exactly on the mean plane are in green; the symmetry elements of the appropriate layer space group are in black. The shaded areas in column (4) cover one unit cell of the corresponding layer space groups. The relatively small figures in this table are reproduced at a larger scale in the supporting information.

0	1	2	3	4
d*x* = 1/2, d*y* = √3/2	[Chem scheme1] 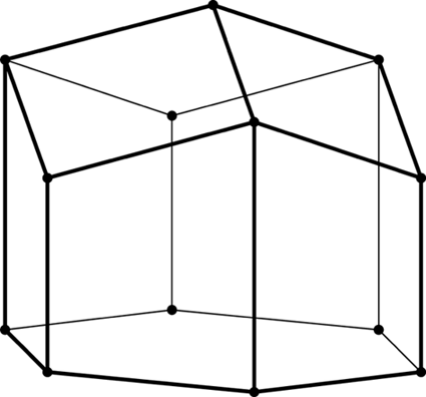	[Chem scheme2] 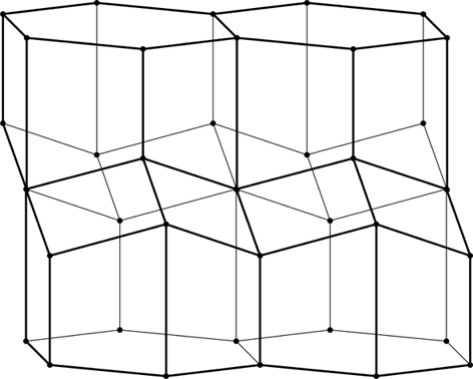	[Chem scheme3] 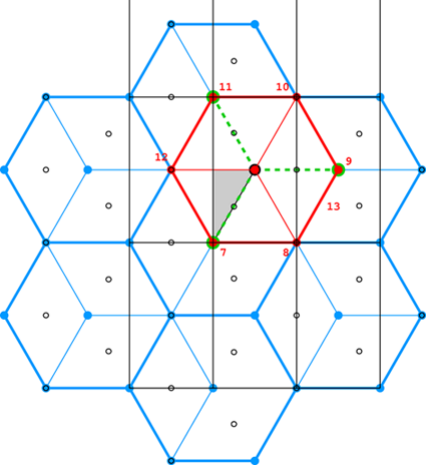	[Chem scheme4] 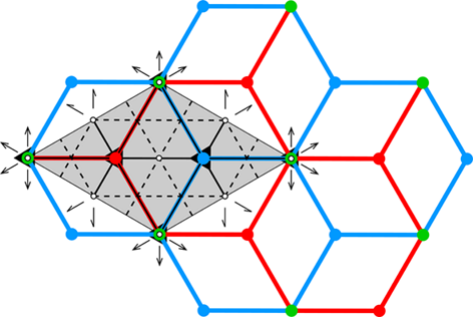
d*x* = 0, d*y* = √3/2	[Chem scheme5] 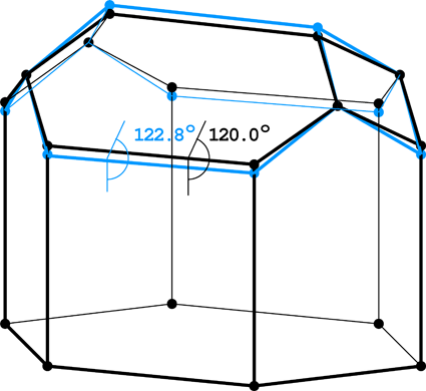	[Chem scheme6] 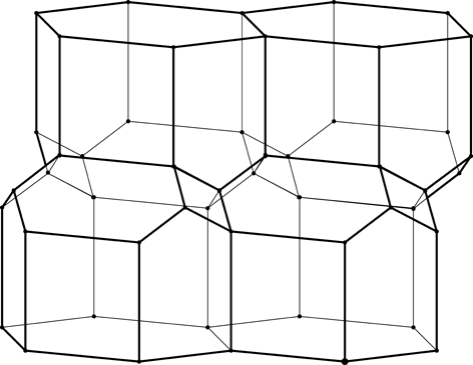	[Chem scheme7] 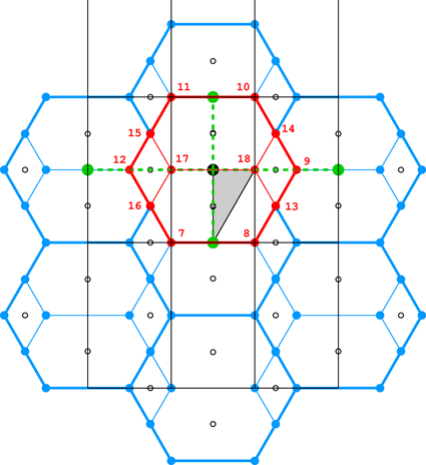	[Chem scheme8] 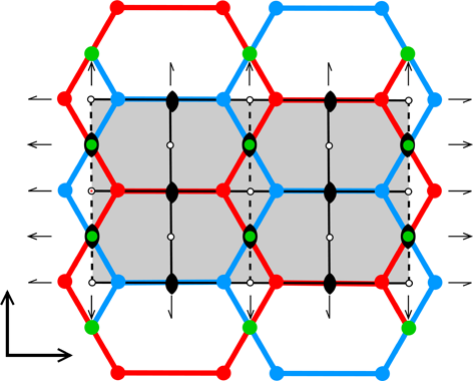
d*x* = 1/4, d*y* = √3/4	[Chem scheme9] 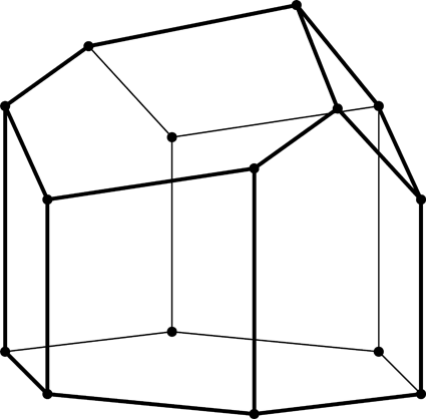	[Chem scheme10] 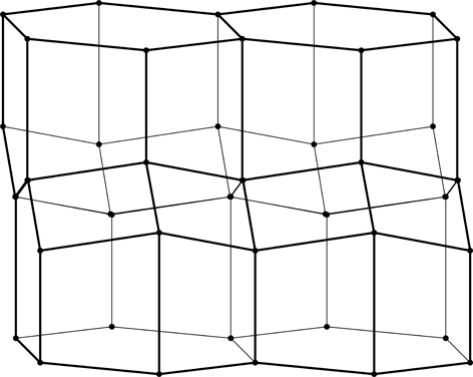	[Chem scheme11] 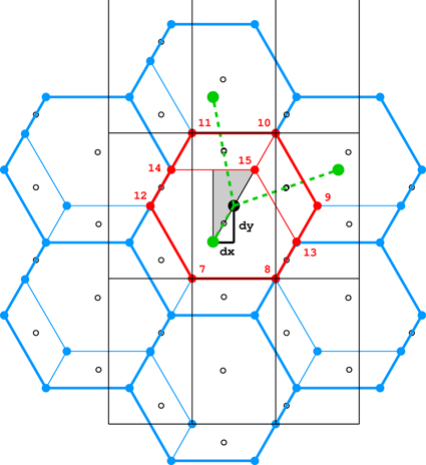	[Chem scheme12] 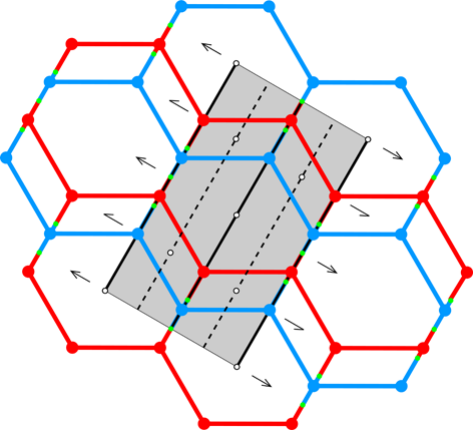
d*x* = 0, d*y* = 0	[Chem scheme13] 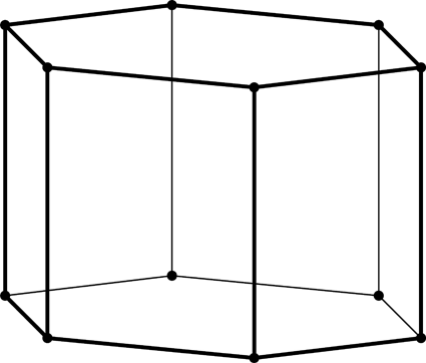	[Chem scheme14] 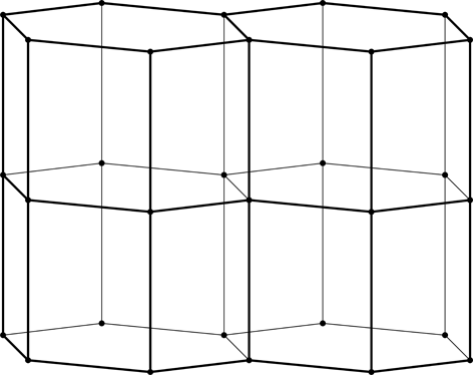	[Chem scheme15] 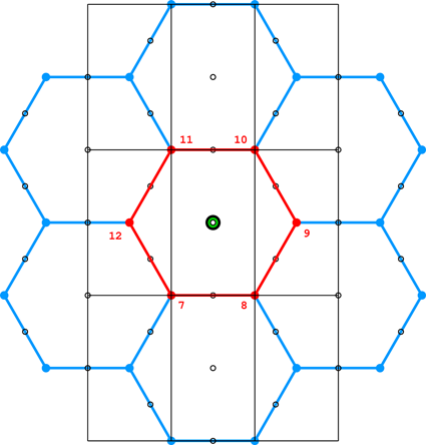	[Chem scheme16] 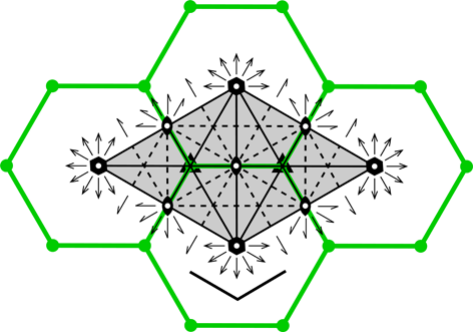
d*x* = 1/4, d*y* = √3/2	[Chem scheme17] 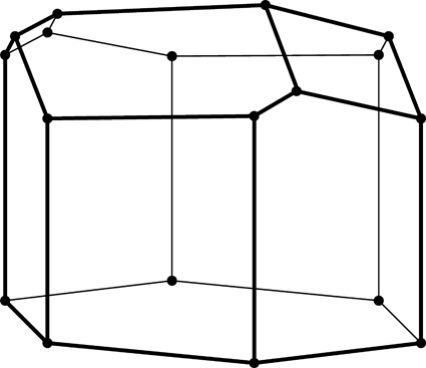	[Chem scheme18] 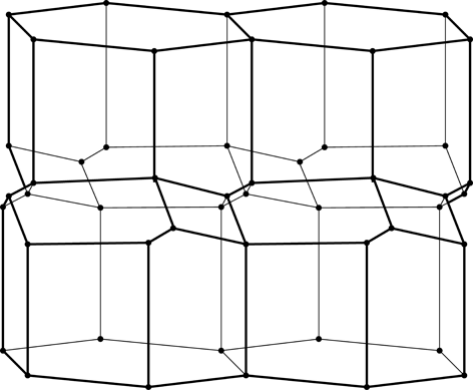	[Chem scheme19] 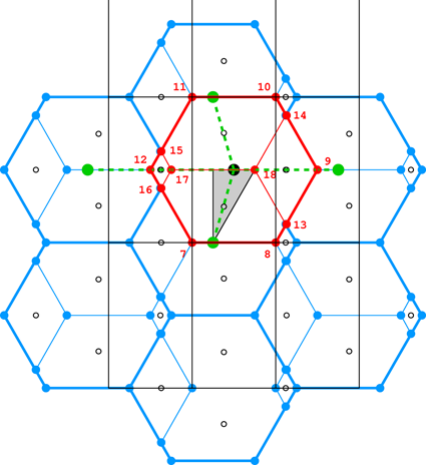	[Chem scheme20] 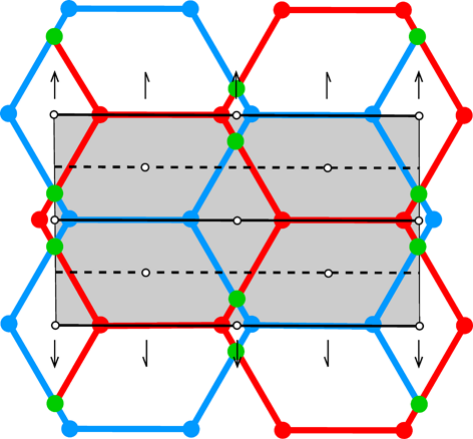
d*x* = 0, d*y* = √3/4	[Chem scheme21] 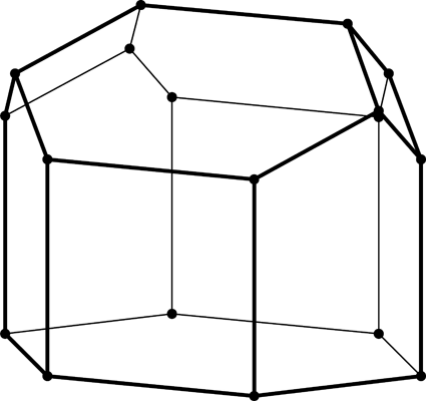	[Chem scheme22] 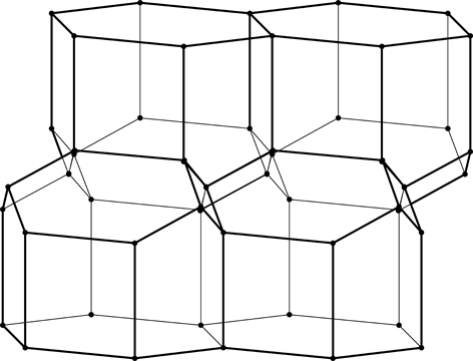	[Chem scheme23] 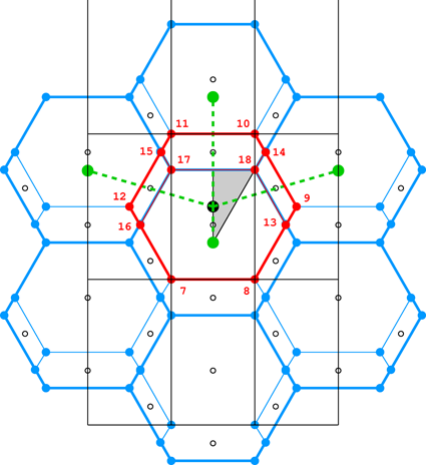	[Chem scheme24] 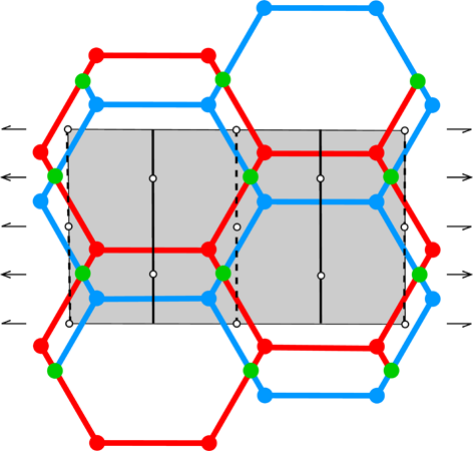
d*x* = 1/5, d*y* = 2√3/5	[Chem scheme25] 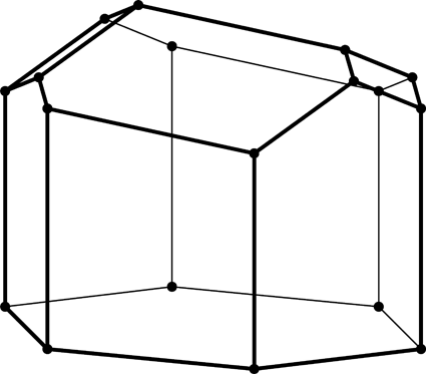	[Chem scheme26] 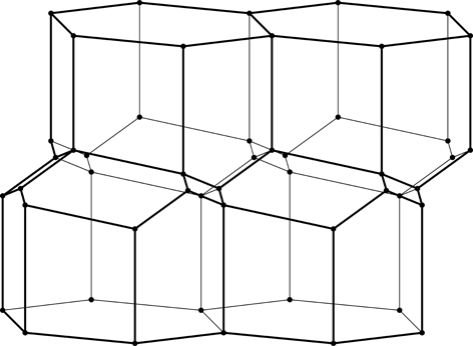	[Chem scheme27] 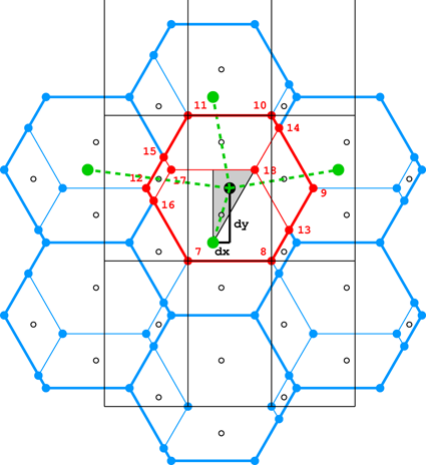	[Chem scheme28] 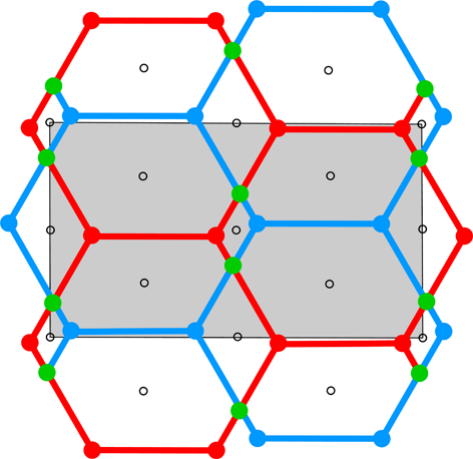

**Table 2:** Characteristics of various honeycombs *A* is the total area of all honeycomb cell faces, Δ*A*/*A* is the relative difference between this area and that of the ‘best’ 6464 honeycomb, expressed as % of the hexagonal cell-base area, equal to 3√3/2 ≃ 2.6, assuming cell depth *d* = 1.5. The Δ*S*/*S* column gives the relative saving of wax, shown as the fraction of a slab of wax (of amount *S*) of thickness *t*/2 and area equal to the cell base. For Δ*S*/*S* calculation the face thickness *t* is assumed as 10% of the unit length of the hexagon edge. The d*x* and d*y* parameters of the bottom entry may adopt any numerical values within the ranges 0 ≤ d*x* ≤ 1/2, 0 ≤ d*y* ≤ d*x*√3/2.

d*x*	d*y*	Topology	Symmetry	*A*	Δ*A*/*A* (%)	*S*	Δ*S*/*S* (%)	Table 1 row
1/2	√3/2	444	*p*3*m*1	11.12132	0.50	0.53083	0.27	1
0	√3/2	6464	*cmma*	11.10843	0	0.53048	0	2 ‘best’
0	√3/2	6464	*cmma*	11.11237	0.15	0.53065	0.13	2 FT
1/4	√3/4	644	*c*2/*m*	11.22592	4.52	0.53564	3.97	3
0	0	6	*p*6/*mmm*	11.59808	18.85	0.55191	16.50	4
1/4	√3/2	6464	*c*2/*m*	11.11442	0.23	0.53070	0.17	5
0	√3/4	6464	*c*2/*m*	11.21222	3.99	0.53528	3.70	6
Any	Any	6464	*p* 1					7
