# Supplementary material for: Honeycombs – their variety, topology and symmetry
Source: Acta Crystallogr A Found Adv. 2025 Feb 14;81(Pt 2):159–66. doi: 10.1107/S2053273325000889 (PMC11873813; doi:10.1107/S2053273325000889)
Supplement: Supplementary file 1 [file a-81-00159-sup1.pdf]

Acta Cryst A FOUNDATIONS ADVANCES

Volume 81 (2025)

Supporting information for article:

Honeycombs – their variety, topology and symmetry

Zbigniew Dauter and Mariusz Jaskolski

1. Fejes Tóth’s derivation of the 6464 honeycomb from the regular octahedron

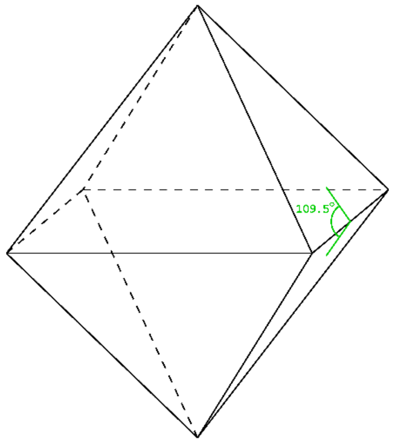

F1

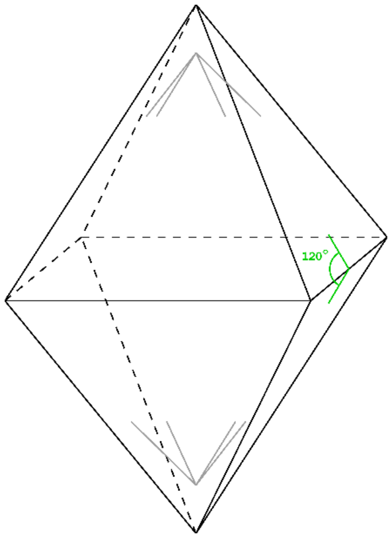

F2

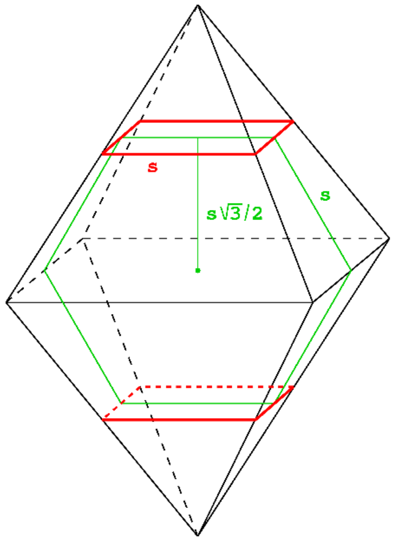

F3

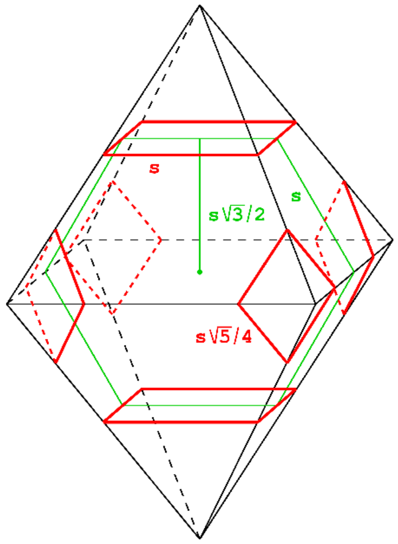

F4

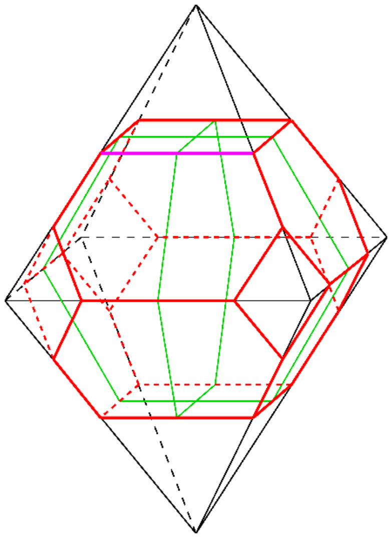

F5

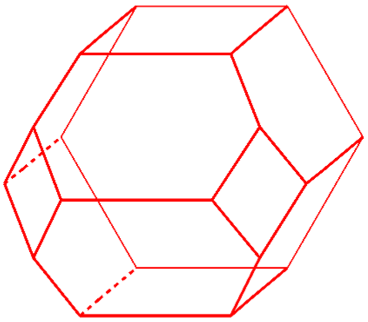

F6

Fejes Tóth starts with the *m*3*m* symmetric octahedron, in which all the dihedral angles between adjacent faces are tetrahedral, i.e. 109.5°, and which has the edge length set arbitrarily at 2s (F1). He then stretches the octahedron along one of its diagonals (vertical in F2) into a tetragonal bipyramid with 2s√3 in height, to obtain the dihedral angles between the upper and lower faces equal to 120.0°. Next, he truncates both ends of the bipyramid at half height, to obtain two new square faces with the edge length of s (F3). The next step is a truncation of the four remaining corners of the bipyramid at 1/4 from the vertices, to obtain four rhombi with s√5/4 as their edge, and s√3/2 and s√2/2 as their diagonals (F4). This leads to the formation of a distorted cuboctahedron (F5) with two hexagonal cross sections (green). Cutting the cuboctahedron in half along such a hexagonal plane and extending the six parallel stump edges will give the honeycomb cell (F6) of Fejes Tóth (1964).

2. Derivation of the 444 honeycomb from the regular octahedron and cube

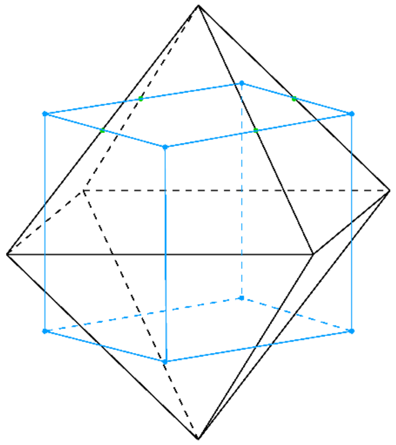

R1

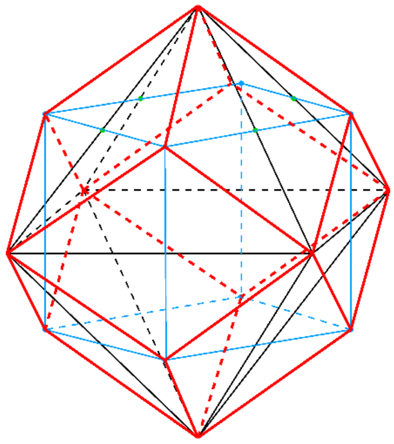

R2

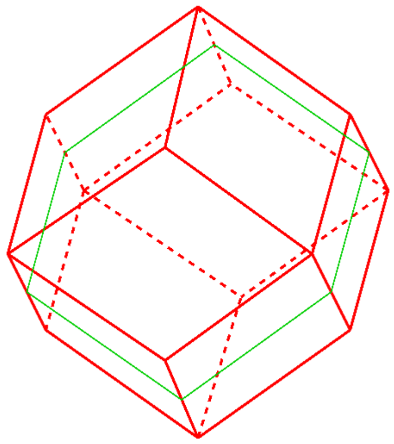

R3

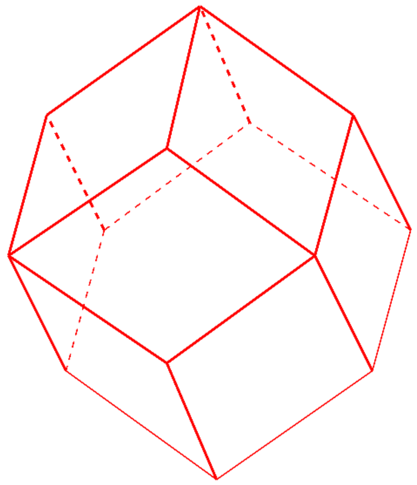

R4

To start with, there are the octahedron (black) and cube (blue) overlapping in such a way that the mid-points of their edges (12 in each case) coincide (R1). Appropriate connections of the protruding vertices (red) form the *m*3*m* symmetric rhombic dodecahedron (R2). Its cross sections perpendicular to (each of) its threefold axis is a symmetric hexagon (R3, green). Cutting the dodecahedron along one of such cross sections and extension of the six parallel stump edges (R4) gives the classic, tri-rhombic 444 honeycomb cell.

3. Calculation of various honeycomb parameters

For convenience, Figs. S2-S8 are reproduced from the main text, together with their extended caption. Each set of figures for a given combination of dx, dy, is followed by a list of vertex coordinates, areas of all unique faces, and the total area of all honeycomb cell faces. The optimum value of the h parameter that minimizes this total area is also given with the relative difference of this area (in %) from the minimum value of the “best” honeycomb, expressed in percent of the hexagonal base area. In these calculations, the percentage values of the “wax economy” assume infinitesimally thin cell walls and therefore do not take into account any real values of their width. The area equations are not given for the last, most general instance of arbitrary dx and dy, since they become exceedingly complicated in that case.

Figs. S2-S8 illustrate certain aspects of honeycombs with various dx, dy layer shifts and, therefore, with various topology and symmetry. In all these figures, panel (a) shows a single cell with all vertices numbered and some dimensions marked in green, and panel (b) shows two pairs of cells from the two layers, illustrating how their bottoms fit together. Panel (c) shows a projection of one upper-layer cell (red) on several lower-layer cells (blue), with the Dirichlet-domain generating points marked green (for the upper cell) and black (lower cells), and with auxiliary vectors between those points (green dash lines) that generate the bottom faces of the red upper cell, also showing the centers of symmetry of the honeycomb as small black circles. The black lines mark the reference frame for the coordinates of the vertices of the red cell (with x axis horizontal, y axis vertical). Panel (d) shows a projection of a fragment of the honeycomb with a few upper- and lower-layer cells, with all edges lying above the mean plane in red and all edges lying below this plane in blue. The elements (vertices and edges) lying exactly on the mean plane are in green. Black symbols show all the symmetry elements of the appropriate layer space group. Panel (e) shows a vertical cross-section of the cell, described by the points marked in black (as in panel a). Some key distances, marked in green in panels (a) and (e), illustrate the relationships between dx, dy and h. These values are used in the calculations presented below. Note that there are no panels S5e and S8e. The former situation is trivial, as all bottom vertices lie at the mean plane with w=0 and the value of h is immaterial. The latter situation is too general and complicated for a simple graphical interpretation.

definition of symbols:

6464 etc.	honeycomb topology expressed as sequence of polygons in the cell base
dx, dy	relative shift of centers of honeycomb cells in the two layers along x and y
cmma etc.	layer space group symmetry in Hermann-Mauguin notation
d	depth of a single cell, *i.e.* half-width of the two-layer honeycomb, assumed d=1.5
w	z-coordinate of honeycomb vertices, *i.e.* their distances from the mean honeycomb plane
A	total area of all faces of a honeycomb cell
h	distance between two planes of Dirichlet generators in two opposite honeycomb layers
h_{opt}	distance h optimized for minimal A for given values of dx, dy
S_{wax}	amount (volume) of wax needed for a single honeycomb cell, assuming the wall thickness t as 10% of the hexagonal base edge, i.e. t=0.1

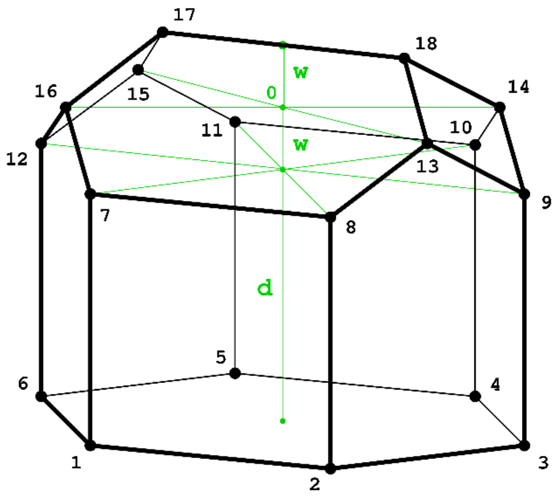

Fig. S2a

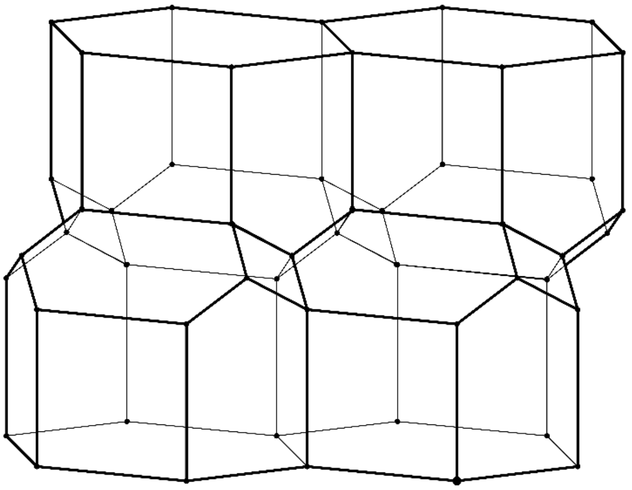

Fig. S2b

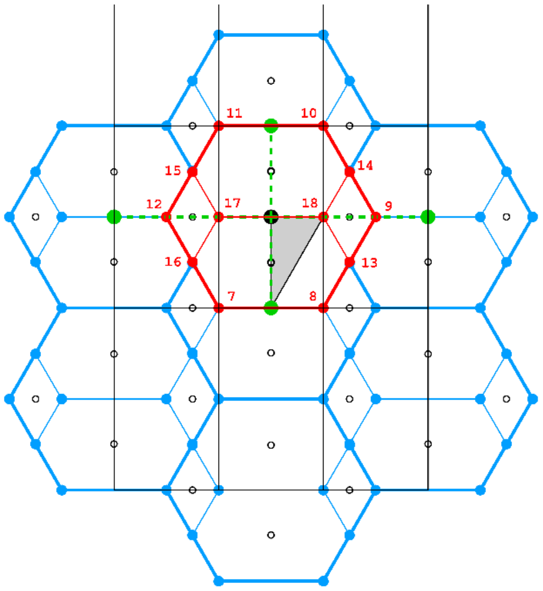

Fig. S2c

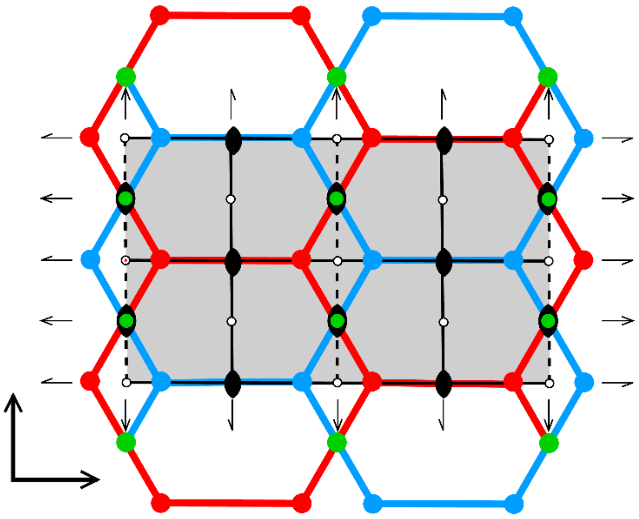

Fig. S2d

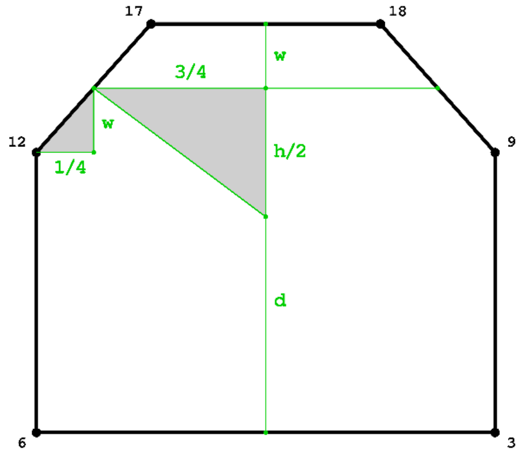

Fig. S2e

6464 **dx = 0** **dy = √3/2** ***cmma*** **Best**

Coordinates of all vertices:

	x	y	z		x	y	z		x	y	z
(1)	-1/2	-√3/2	-d	(7)	-1/2	-√3/2	-w	(13)	3/4	-√3/4	0
(2)	1/2	-√3/2	-d	(8)	1/2	-√3/2	-w	(14)	3/4	√3/4	0
(3)	1	0	-d	(9)	1	0	-w	(15)	-3/4	√3/4	0
(4)	1/2	√3/2	-d	(10)	1/2	√3/2	-w	(16)	-3/4	-√3/4	0
(5)	-1/2	√3/2	-d	(11)	-1/2	√3/2	-w	(17)	-1/2	0	w
(6)	-1	0	-d	(12)	-1	0	-w	(18)	1/2	0	w

Lengths of relevant edges (distances):

(1-2)	1	1.0	6x(1-7)	120°
(1-7)	d - w	1.220786	2x(7-8)	122.81°
(7-17)	$\sqrt{(3/4 + 4w^2)}$	1.030460	4x(8-13)	105.72°
(13-16)	3/2	1.5	4x(9-13)	130.18°
(9-18)	$\sqrt{(1/4 + 4w^2)}$	0.749564	4x(13-18)	124.10°
(13-14)	√3/2	0.866025	1(17-18)	114.37°
(8-13)	$\sqrt{(1/4 + w^2)}$	0.572679		
	Total	17.196852	Average	120°

Areas of representative faces:

(1-2-8-7)	d - w	1.220786
(8-9-13)	w/2	0.139607
(7-8-13-18-17-16)	$5*\sqrt{(3/4 + 4w^2)}/4$	1.288071
(9-13-18-14)	$\sqrt{(3/4 + 12w^2)}/4$	0.324569

Total area of all faces (except the hexagonal base):

A = 6*(1-2-8-7) + 4*(8-9-13) + 4*(7-8-13-16) + 2*(9-13-18-14) =

$= 6*(d-w) + 2w + 5\sqrt{(3/4 + 4w^2)}/2 + \sqrt{(3/4+ 12w^2)}/2$

Partial derivative of A with respect to variable w:

$\partial A/\partial w = -4 + 10w/\sqrt{(3/4 + 4w^2)} + 6w/\sqrt{(3/4 + 12w^2)} = 0$

for d = 1.5 w = 0.279214 h_{opt} = 1.343057 A = 11.108426

$w*h_{opt}$ = 3/8 °A/A = 0.0 %

for t = 0.1 S_{wax} = 0.530476

6464 **dx = 0** **dy = √3/2** ***cmma*** **Fejes Tóth**

Coordinates of all vertices:

	x	y	z		x	y	z		x	y	z
(1)	-1/2	-√3/2	-d	(7)	-1/2	-√3/2	-w	(13)	3/4	-√3/4	0
(2)	1/2	-√3/2	-d	(8)	1/2	-√3/2	-w	(14)	3/4	√3/4	0
(3)	1	0	-d	(9)	1	0	-w	(15)	-3/4	√3/4	0
(4)	1/2	√3/2	-d	(10)	1/2	√3/2	-w	(16)	-3/4	-√3/4	0
(5)	-1/2	√3/2	-d	(11)	-1/2	√3/2	-w	(17)	-1/2	0	w
(6)	-1	0	-d	(12)	-1	0	-w	(18)	1/2	0	w

Lengths of relevant edges (distances):

(1-2)	1	1.0	6x(1-7)	120°
(1-7)	d - w	1.25	2x(7-8)	120°
(7-17)	$\sqrt{3/4 + 4w^2}$	1.0	4x(8-13)	104.48°
(13-16)	3/2	1.5	4x(9-13)	127.76°
(9-18)	$\sqrt{1/4 + 4w^2}$	0.707107	4x(13-18)	127.76°
(13-14)	√3/2	0.866025	1x(17-18)	120°
(8-13)	$\sqrt{1/4 + w^2}$	0.559017		
	Total	17.208204	Average	120°

Areas of representative faces:

(1-2-8-7)	d - w	1.25
(8-9-13)	w/2	0.125
(7-8-13-18-17-16)	$5\sqrt{3/4 + 4w^2}/4$	1.25
(9-13-18-14)	$\sqrt{3/4 + 12w^2}/4$	0.306186

Total area of all faces (except the hexagonal base):

A = 6*(1-2-8-7) + 4*(8-9-13) + 4*(7-8-13-16) + 2*(9-13-18-14) =

$= 6(d-w) + 2w + 5\sqrt{3/4 + 4w^2}/2 + \sqrt{3/4+ 12w^2}/2$

for	d = 1.5	w = 0.25	h = 1.5	A = (42+√6)/4
		w*h = 3/8	°A/A = 0.15 %	A = 11.112372
for	t = 0.1	S_{wax} = 0.530651		

444 **dx = 1/2** **dy = √3/2** ***p*$\bar{3}$*m*1** **classic**

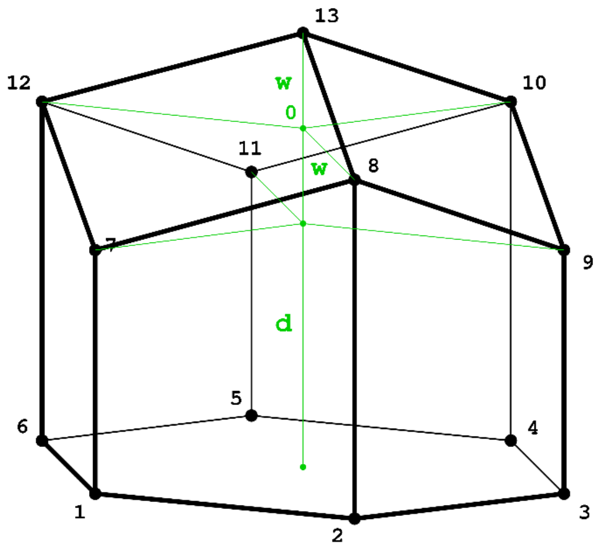

Fig. S3a

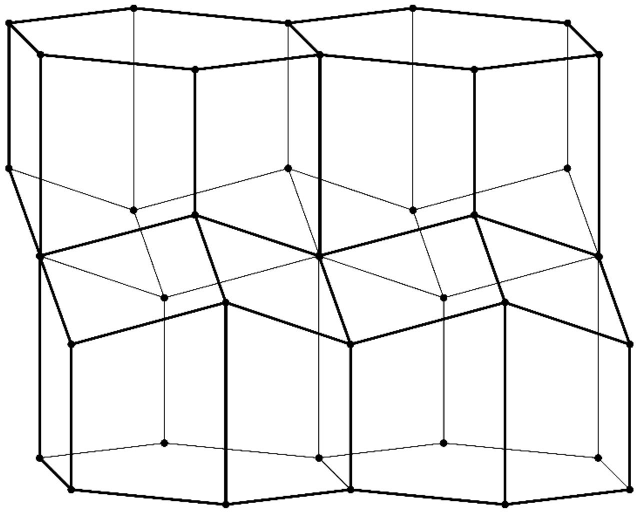

Fig. S3b

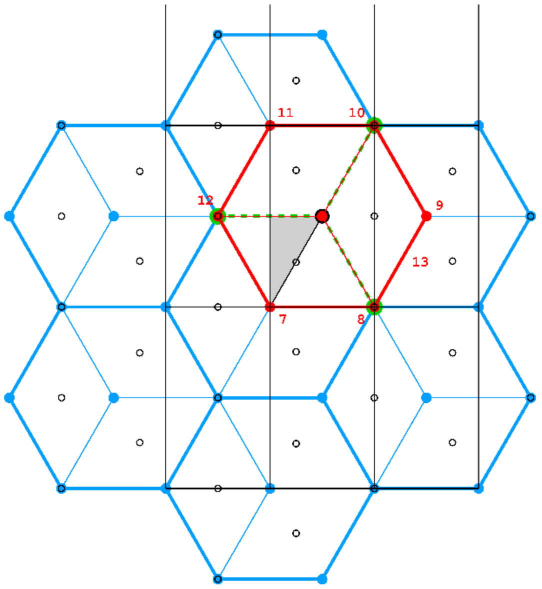

Fig. S3c

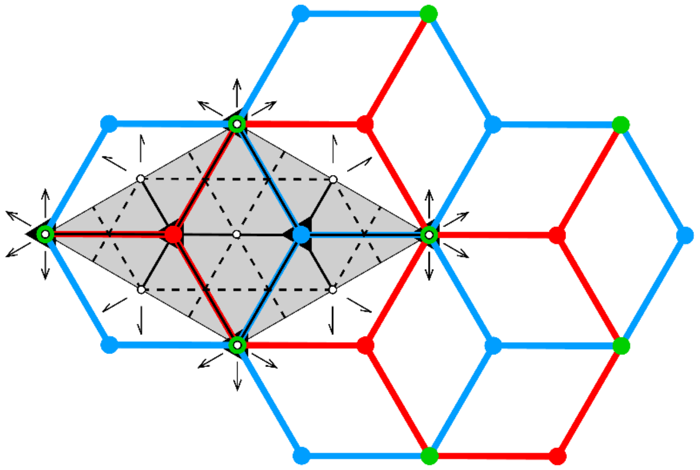

Fig. S3d

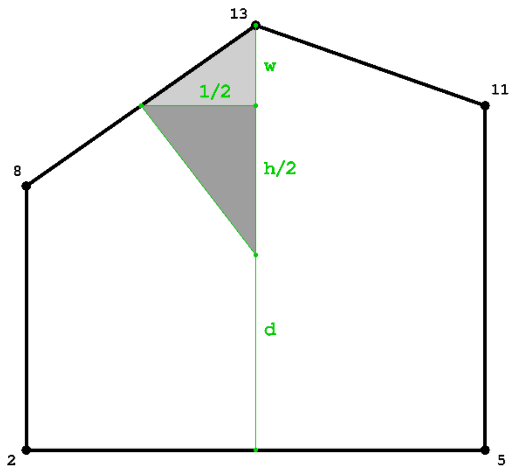

Fig. S3e

444 **dx = 1/2** **dy = √3/2** ***p*3̄*m*1** **classic**

Coordinates of all vertices:

	x	y	z		x	y	z		x	y	z
(1)	-1/2	-√3/2	-d	(5)	-1/2	√3/2	-d	(9)	1/2	0	-w
(2)	1/2	-√3/2	-d	(6)	-1	0	-d	(10)	-1/2	√3/2	0
(3)	1	0	-d	(7)	-1/2	-√3/2	-w	(11)	0	√3/2	-w
(4)	1/2	√3/2	-d	(8)	1/2	-√3/2	0	(12)	-1/2	0	0
								(13)	0	0	w

Lengths of relevant edges (distances):

(1-2)	1	1.0	6x(1-7)	120°
(1-7)	d - w	1.146447	6x(7-8)	120°
(2-8)	d	1.5	3x(8-13)	120°
(7-8)	$\sqrt{(3 + 12w^2)}/2$	1.060660		
(7-13)	$\sqrt{(1 + 4w^2)}/2$	1.224744	Average	120°
(8-12)	√3	1.732051		
	Total	17.485281		

Areas of representative faces:

(1-2-8-7)	d – w/2	1.323223
(7-8-13-12)	$\sqrt{(3 + 12w^2)}/2$	1.060660

Total area of all faces (except the hexagonal base):

$A = 6 * (1\text{-}2\text{-}7\text{-}8) + 3 * (7\text{-}8\text{-}13\text{-}12) = 6z – 3w + 3\sqrt{(3 + 12w^2)}/2$

Partial derivative of A with respect to variable w:

$\partial A/\partial w = -3 + 18w/\sqrt{(3 + 12w^2)} \quad = 0$

for	d = 1.5	w = √2/4	h = √2	A = 9+√18/2
		0.353553	1.414214	11.121320
		$w*h_{opt} = 1/2$	°A/A = 0.50 %	
for	t = 0.1	$S_{wax} = 0.530828$		

644 **dx = 1/4** **dy = $\sqrt{3}/4$** ***c*2/*m***

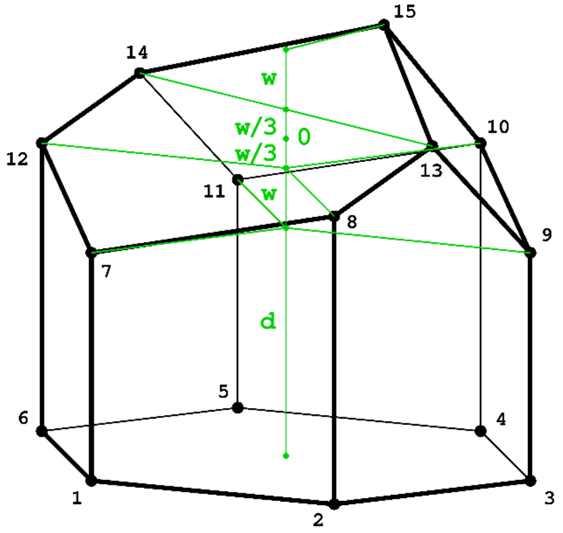

Fig. S4a

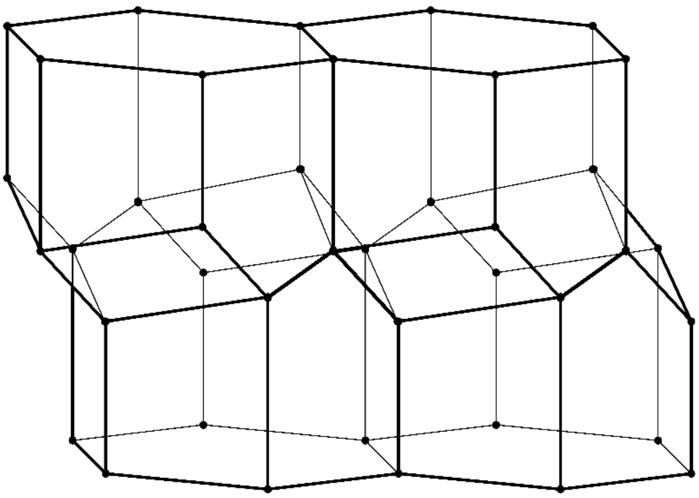

Fig. S4b

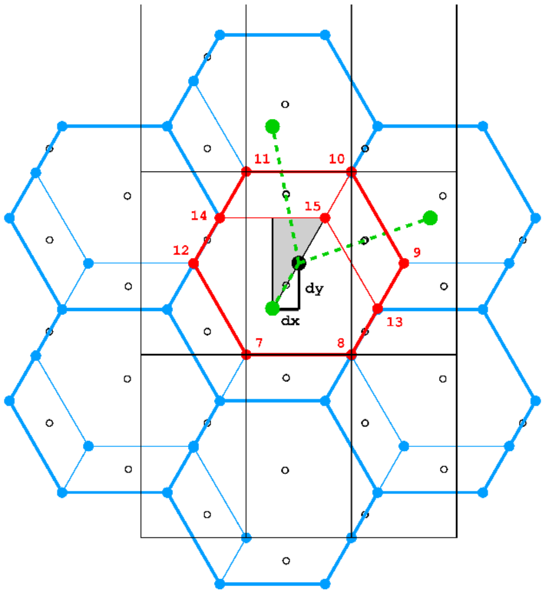

Fig. S4c

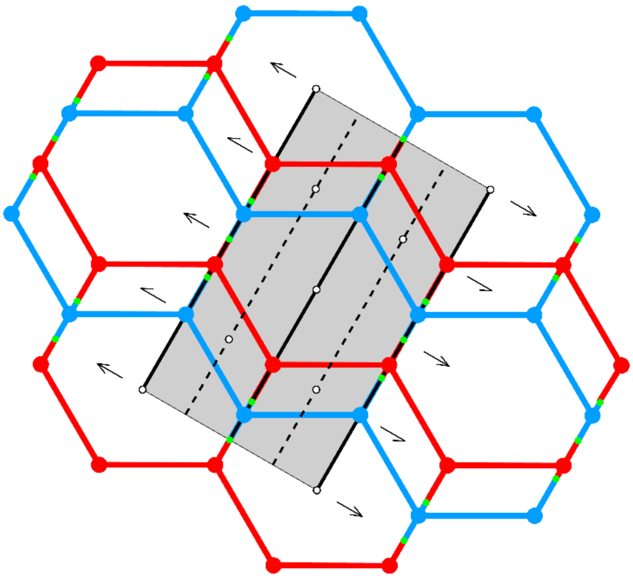

Fig. S4d

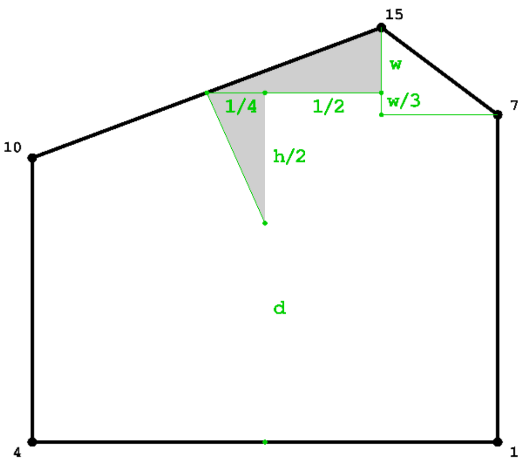

Fig. S4e

644 dx = 1/4 dy = √3/4 *c*2/*m*

Coordinates of all vertices:

	x	y	z		x	y	z		x	y	z
(1)	-1/2	-√3/2	-d	(7)	-1/2	-√3/2	-w	(13)	3/4	-√3/4	w/3
(2)	1/2	-√3/2	-d	(8)	1/2	-√3/2	-w/3	(14)	-3/4	√3/4	w/3
(3)	1	0	-d	(9)	1	0	-w	(15)	1/4	√3/4	w
(4)	1/2	√3/2	-d	(10)	1/2	√3/2	-w/3				
(5)	-1/2	√3/2	-d	(11)	-1/2	√3/2	-w				
(6)	-1	0	-d	(12)	-1	0	-w/3				

Lengths of relevant edges (distances):

(1-2)	1	1.0	6x(1-7)	120°
(1-7)	d - w	1.212825	2x(7-8)	108.04°
(2-8)	d - w/3	1.404275	2x(8-13)	90°
(7-8)	$\sqrt{(9 + 4w^2)/3}$	1.018162	2x(9-13)	117.77°
(8-13)	$\sqrt{(9/4 + 4w^2)/3}$	0.535400	2x(9-10)	134.34°
(9-13)	$\sqrt{(9/4 + 16w^2)/2}$	0.629772	2x(13-15)	117.63°
(7-15)	$\sqrt{(9/4+4w^2)}$	1.606200	1x(10-15)	124.46°
(8-12)	√3	1.732051	Average	116.47° = 1980/17
	Total	16.920393		

Areas of representative faces:

(1-2-8-7)	d - 2w/3	1.308552
(8-9-13)	w/2	0.143587
(9-10-15-13)	$\sqrt{(3/16 + 7w^2/3)}$	0.616380
(7-8-13-15-14-12)	$\sqrt{(3 + 16w^2/3)}$	1.854677

Total area of all faces (except the hexagonal base):

A = 6*(1-2-8-7) + 2*(8-9-13) + 2*(9-10-15-13) + (7-8-13-15-14-12) =

$$= 6d - 3w + \sqrt{(3+16w^2/3)} + \sqrt{(3/4 + 28w^2/3)}$$

Partial derivative of A with respect to variable w:

$$\partial A/\partial w = -3 + (16/3)w/\sqrt{(3+16w^2/3)} + (28/3)w/\sqrt{(3/4 + 28w^2/3)} = 0$$

for d = 1.5 w = 0.287175 h_{opt} = 1.305828 A = 11.225922

$w{*}h_{opt} = 3/8$ °A/A = 4.52 %

for t = 0.1 S_{wax} = 0.535636

6 **dx = 0** **dy = 0** ***p6/mmm***

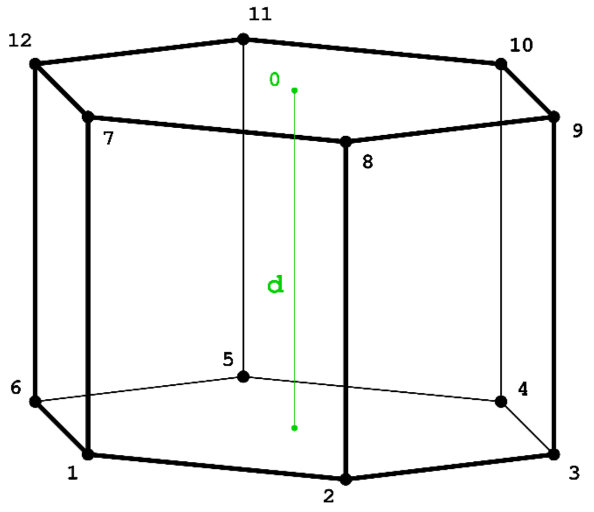

Fig. S5a

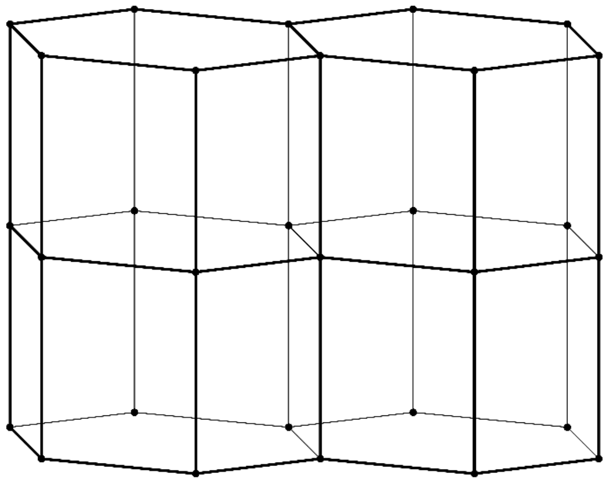

Fig. S5b

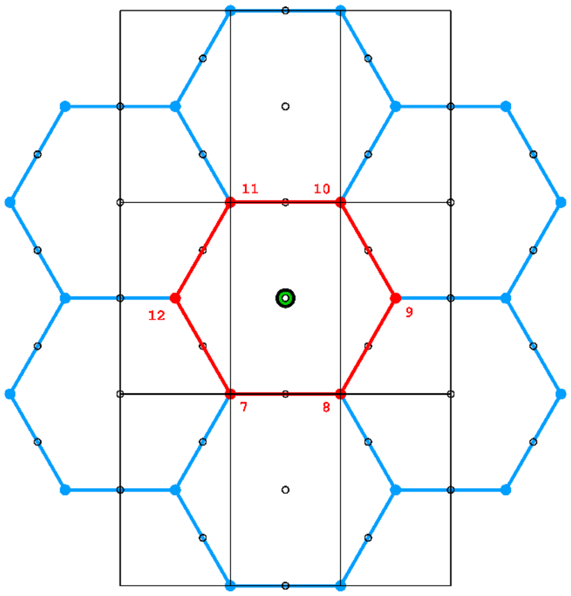

Fig. S5c

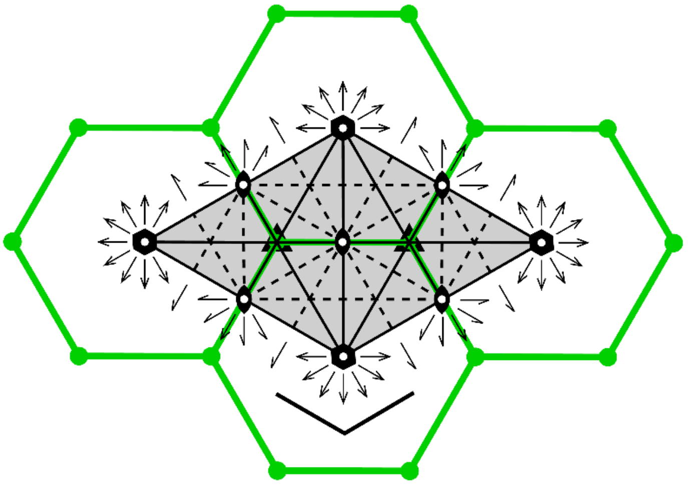

Fig. S5d

6 **dx = 0** **dy = 0** ***p*6/*mmm***

Coordinates of all vertices:

	x	y	z		x	y	z		x	y	z
(1)	-1/2	-√3/2	-d	(5)	-1/2	√3/2	-d	(9)	1/2	0	0
(2)	1/2	-√3/2	-d	(6)	0	0	-d	(10)	-1/2	√3/2	0
(3)	1	0	-d	(7)	-1/2	-√3/2	0	(11)	0	√3/2	0
(4)	1/2	√3/2	-z	(8)	1/2	-√3/2	0	(12)	-1/2	0	0

Lengths of relevant edges (distances):

(1-2)	1	1.0	6x(1-7)	120°
(1-7)	d	1.5	6x(7-8)	90°
	Total	15.0	Average	105°

Areas of representative faces:

(1-2-8-7)	d	1.5
(8-9-13)	3√3/2	2.598076

Total area of all faces (except the hexagonal base):

A = 6 * (1-2-7-8) + (7-8-9-10-11-12) = 6d + 3√3/2

for z = 1 dx = 0 dy = 0 w = 0 h = ? A = 9+3√3/2

°A/A = 18.85 % A = 11.598076

for t = 0.1 S_{wax} = 0.551913

6464 **dx = 1/4** **dy = √3/2** ***c*2/*m***

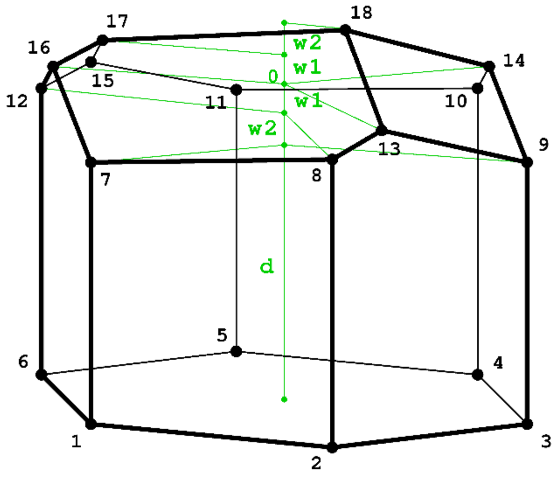

Fig. S6a

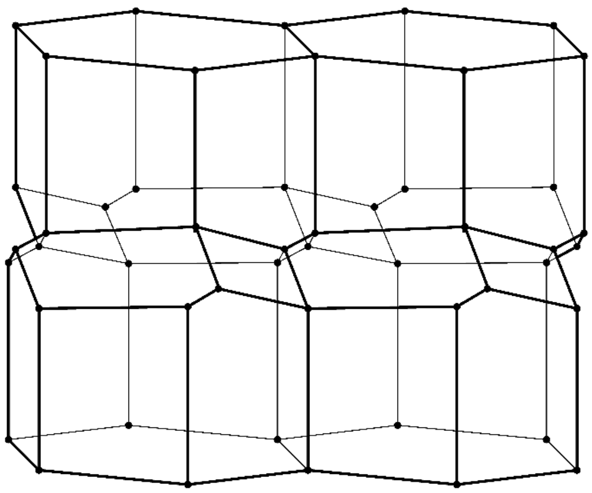

Fig. S6b

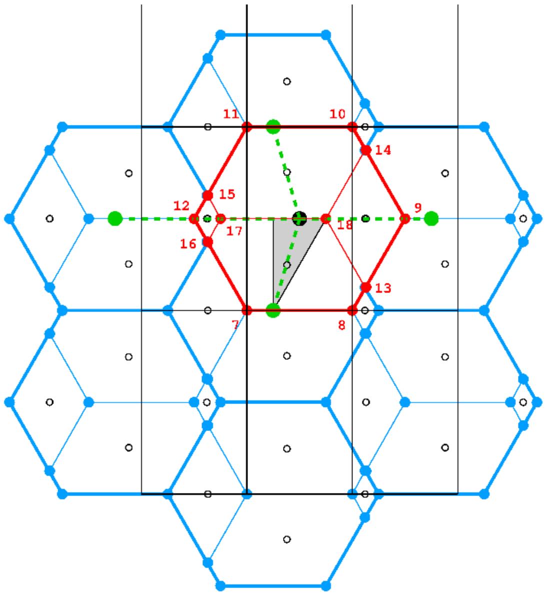

Fig. S6c

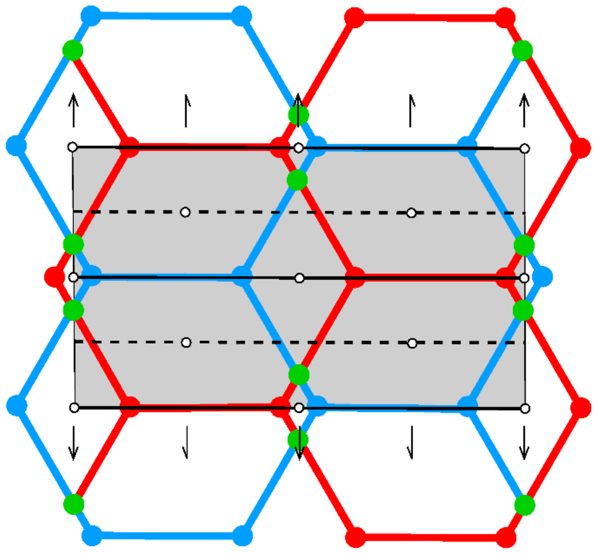

Fig. S6d

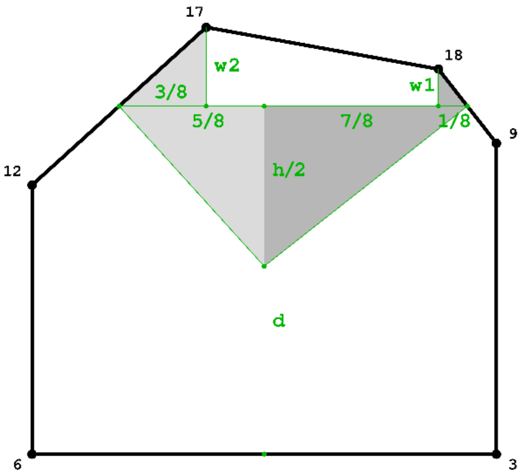

Fig. S6e

6464 **dx = 1/4** **dy = √3/2** ***c*2/*m***

Coordinates of all vertices:

	x	y	z		x	y	z		x	y	z
(1)	-1/2	-√3/2	-z	(7)	-1/2	-√3/2	-15w	(13)	5/8	-3√3/8	0
(2)	1/2	-√3/2	-z	(8)	1/2	-√3/2	-7w	(14)	5/8	3√3/8	0
(3)	1	0	-z	(9)	1	0	-15w	(15)	-7/8	√3/8	0
(4)	1/2	√3/2	-z	(10)	1/2	√3/2	-7w	(16)	-7/8	-√3/8	0
(5)	-1/2	√3/2	-z	(11)	-1/2	√3/2	-15w	(17)	-3/4	0	7w
(6)	-1	0	-z	(12)	-1	0	-7w	(18)	3/4	0	15w

Lengths of relevant edges (distances):

(1-2)	1	1.0	(1-7)	120°
(1-7)	d - 15w	1.159276	(7-8),(10-11)	121.77°
(2-8)	d - 7w	1.340995	(8-13),(10-14)	97.56°
(7-8)	$\sqrt{(1 + 64w^2)}$	1.016377	(9-13),(9-14)	125.62°
(8-13)	$\sqrt{(1/16 + 49w^2)}$	0.296281	(13-18),(14-18)	121.12°
(9-13)	$\sqrt{(9/16 + 225w^2)}$	0.823768	(7-16),(11-15)	113.26°
(7-18)	$\sqrt{(37/16 + 900w^2)}$	1.332994	(12-15),(12-16)	132.91°
(8-16)	$\sqrt{(37/16 + 49w^2)}$	1.528981	(15-17),(16-17)	129.53°
			(17-18)	116.46°
	Total	17.270236	Average	120°

Areas of representative faces:

(1-2-8-7)	d - 11w	1.250139
(8-9-13)	9w/2	0.102216
(9-13-18-14)	$9*\sqrt{(3/64+75w^2)}/4$	0.658185
(12-15-17-16)	$\sqrt{(3/64+147w^2)}/4$	0.087579
(7-16-17-18-13-8)	$19*\sqrt{(3/64+39w^2)}/4$	1.229483

Total area of all faces (except the hexagonal base):

A = 6*(1-2-8-7) + 4*(8-9-13) + 2*(7-8-13-18-17-16) + (9-13-19-14) + (12-15-17-16) =

$$= 6d - 48w + 19*\sqrt{(3/64+39w^2)}/2 + \sqrt{(3/64+147w^2)}/4 + 9*\sqrt{(3/64+75w^2)}/4$$

Partial derivative of A with respect to variable w:

$$\partial A/\partial w = -48 + 741w/\sqrt{(3/64+39w^2)}/2 + 147w/\sqrt{(3/64+147w^2)}/4 + + 675w/\sqrt{(3/64+75w^2)}/4 = 0$$

for z = 1	7w = 0.159005 =	7w*h = 7/32	h_{opt} = 1.375745
	15w = 0.340724 =	15w*h = 15/32	A = 11.114423
	w = 0.0227150	w*h = 1/32	°A/A = 0.23 %

for t = 0.1 S_{wax} = 0.530696

6464 **dx = 0** **dy = √3/4** ***c*2/*m***

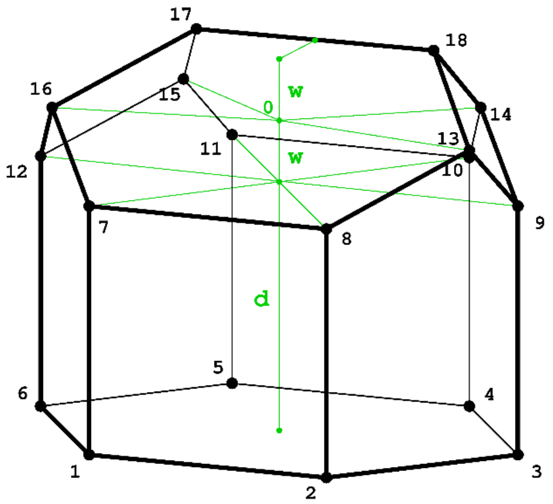

Fig. S7a

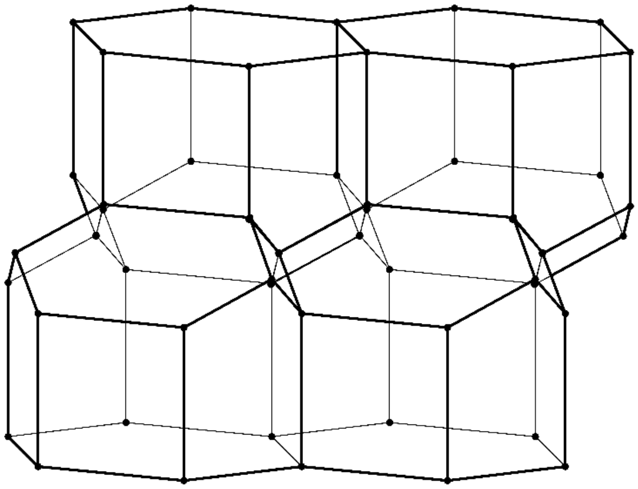

Fig. S7b

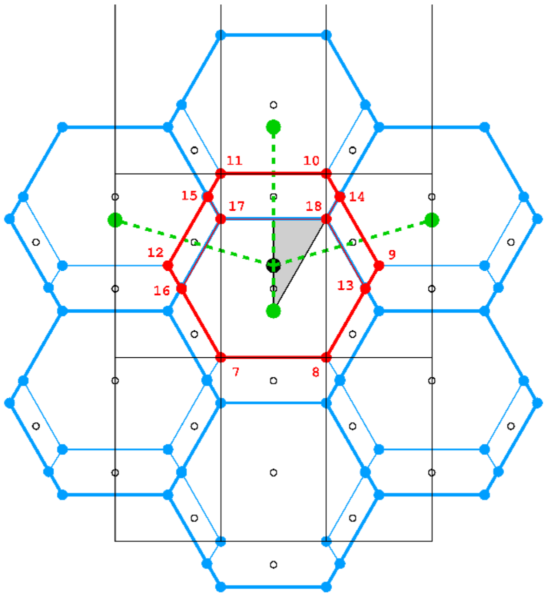

Fig. S7c

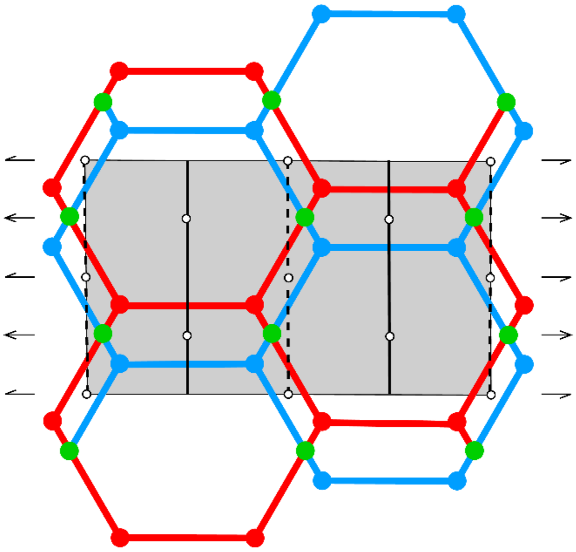

Fig. S7d

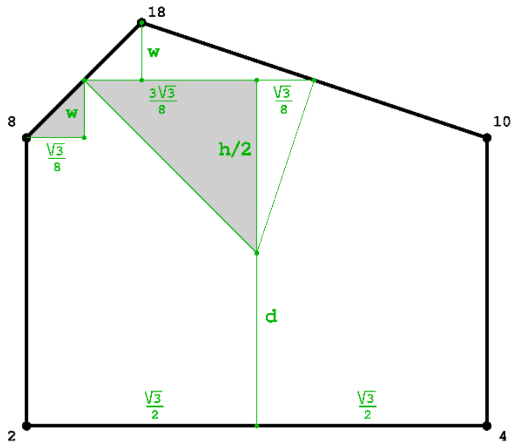

Fig. S7e

6464 dx = 0 dy = √3/4 *c*2/*m*

Coordinates of all vertices:

	x	y	z		x	y	z		x	y	z
(1)	-1/2	-√3/2	-d	(7)	-1/2	-√3/2	-w	(13)	7/8	-√3/8	0
(2)	1/2	-√3/2	-d	(8)	1/2	-√3/2	-w	(14)	5/8	3√3/8	0
(3)	1	0	-d	(9)	1	0	-w	(15)	-5/8	3√3/8	0
(4)	1/2	√3/2	-d	(10)	1/2	√3/2	-w	(16)	-7/8	-√3/8	0
(5)	-1/2	√3/2	-d	(11)	-1/2	√3/2	-w	(17)	-1/2	√3/4	w
(6)	-1	0	-d	(12)	-1	0	-w	(18)	1/2	√3/4	w

Lengths of relevant edges (distances):

(1-2)	1	1.0	(1-7)	120°
(1-7)	d - w	1.267330	(7-8)	109.71°
(8-13)	$\sqrt{9/16 + w^2}$	0.785261	(8-13),(7-16)	99.71°
(9-13)	$\sqrt{1/16 + w^2}$	0.341519	(9-13),(12-16)	123.25°
(7-17)	$\sqrt{27/16 + 4w^2}$	1.379870	(9-14),(12-15)	140.13°
(13-16)	7/4	1.75	(10-14),(11-15)	111.47°
(11-17)	$\sqrt{3/16 + 4w^2}$	0.635642	(10-11)	137.06°
(14-15)	5/4	1.25	(13-18),(16-17)	120.16°
			(14-18),(15-17)	125.28°
			(17-18)	113.23°
	Total	17.364663	Average	120°

Areas of representative faces:

(1-2-8-7)	d - w	1.267333
(8-9-13)	w/2	0.116333
(9-13-18-14)	$\sqrt{27/64+13w^2}/4$	0.265237
(7-8-13-18-17-16)	$11\sqrt{27/16+4w^2}/8$	1.897318
(10-11-15-17-18-14)	$9\sqrt{3/16+4w^2}/8$	0.715092

Total area of all faces (except the hexagonal base):

A = 6*(1-2-8-7) + 4*(8-9-13) + 2*(9-13-18-14) + (7-8-13-18-17-16) + (10-11-15-17-18-14) =

$= 6d - 4w + \sqrt{27/64 + 13w^2}/2 + 11\sqrt{27/16 + 4w^2}/8 + 9\sqrt{3/16 + 4w^2}/8$

Partial derivative of A with respect to variable w:

$\partial A/\partial w = -4 + (13/2)w/\sqrt{27/64 + 13w^2} + (11/2)w/\sqrt{27/16 + 4w^2} + (9/2)w/\sqrt{3/16 + 4w^2}$

for	d = 1.5	w = 0.232670	h_{opt} = 1.208795	A = 11.212218
$w*h_{opt}$ = 9/32		w = 349/1500	h_{opt} = 3375/2792	°A/A = 3.99 %
for	t = 0.1	S_{wax} = 0.535280		

6464 **dx = ?** **dy = ?** $\boldsymbol{p}\bar{1}$

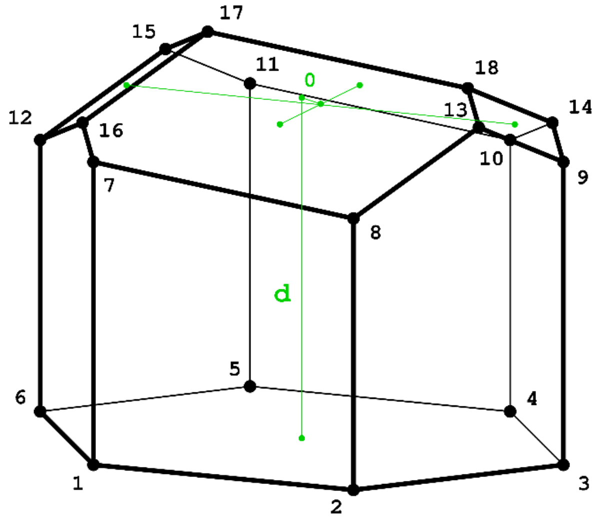

Fig. S8a

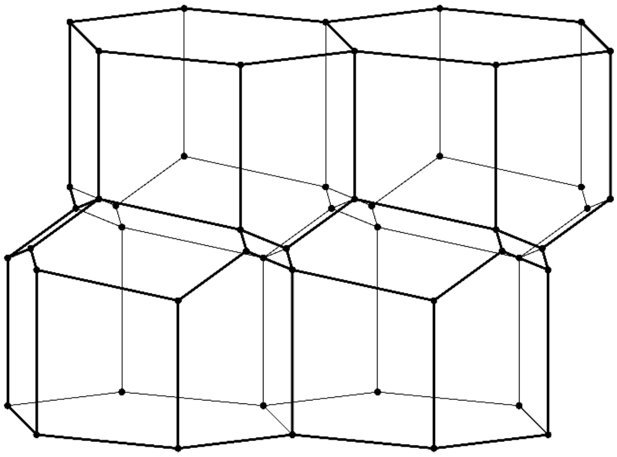
Fig. S8b

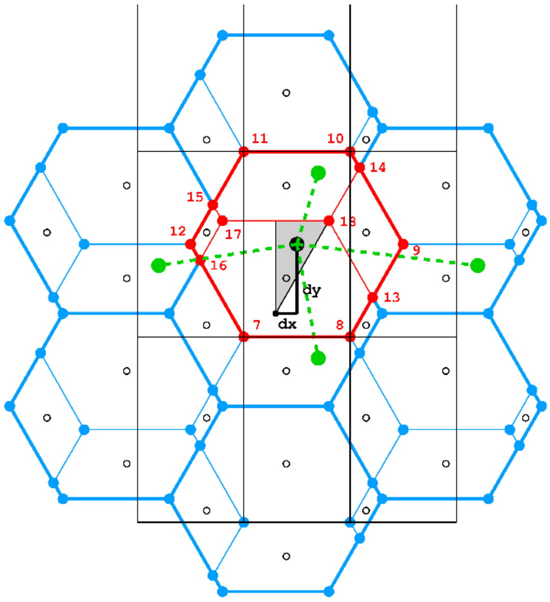

Fig. S8c

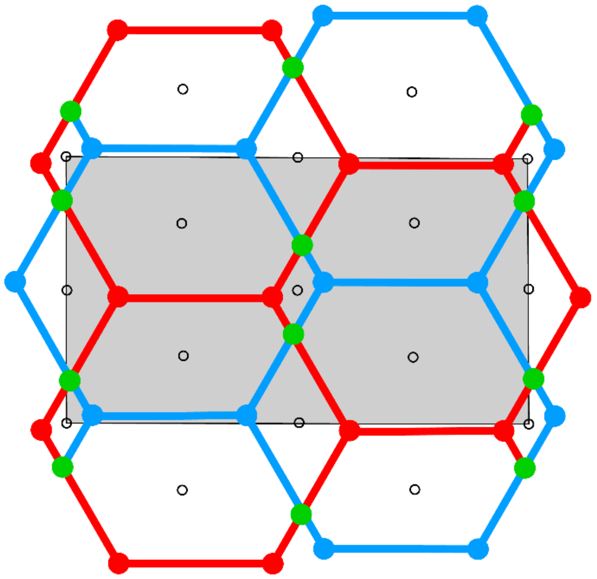
Fig. S8d

6464 **dx = ?** **dy = ?** ***p*$\bar{1}$**

Coordinates of all vertices:

	x	y	z
(1)	-1/2	-√3/2	-d
(2)	1/2	-√3/2	-d
(3)	1	0	-d
(4)	1/2	√3/2	-d
(5)	-1/2	√3/2	-d
(6)	-1	0	-d
(7)	-1/2	-√3/2	$-dx/(2h)+dx^2/(2h)-\sqrt{3}dy/(2h)+dy^2/(2h)$
(8)	1/2	-√3/2	$dx/(2h)+dx^2/(2h)-\sqrt{3}dy/(2h)+dy^2/(2h)$
(9)	1	0	$-dx/(2h)+dx^2/(2h)-\sqrt{3}dy/(2h)+dy^2/(2h)$
(10)	1/2	√3/2	$dx/(2h)+dx^2/(2h)-\sqrt{3}dy/(2h)+dy^2/(2h)$
(11)	-1/2	√3/2	$-dx/(2h)+dx^2/(2h)-\sqrt{3}dy/(2h)+dy^2/(2h)$
(12)	-1	0	$dx/(2h)+dx^2/(2h)-\sqrt{3}dy/(2h)+dy^2/(2h)$
(13)	1-dx/2-dy√3/6	dx√3/2+dy/2	dx/h-2dxdy/(√3h)
(14)	1/2-dx/2+dy√3/6	√3/2+dx√3/2-dy/2	-dx/h+2dxdy/(√3h)
(15)	-1/2-dx/2-dy√3/6	√3/2-dx√3/2-dy/2	dx/h-2dxdy/(√3h)
(16)	-1-dx/2+dy√3/6	-dx√3/2+dy/2	-dx/h+2dxdy/(√3h)
(17)	-1/2-dx	√3/2-dy	$-dx/(2h)-dx^2/(2h)+\sqrt{3}dy/(2h)-dy^2/(2h)$
(18)	1/2-dx	√3/2-dy	$dx/(2h)-dx^2/(2h)+\sqrt{3}dy/(2h)-dy^2/(2h)$

4.

Reference

Fejes Tóth, L. (1964). *Bull. Amer. Math. Soc.* **70**, 468-481.
